# Pulmonary vascular and right ventricular dysfunction in adult critical care: current and emerging options for management: a systematic literature review

**DOI:** 10.1186/cc9264

**Published:** 2010-09-21

**Authors:** Laura C Price, Stephen J Wort, Simon J Finney, Philip S Marino, Stephen J Brett

**Affiliations:** 1 Department of Critical Care, National Heart and Lung Institute, Imperial College London, Royal Brompton Hospital, Sydney Street, London SW3 6NP, UK; 2 Centre for Perioperative Medicine and Critical Care Research, Imperial College Healthcare NHS Trust, Hammersmith Hospital, Du Cane Road, London W12 0HS, UK

## Abstract

**Introduction:**

Pulmonary vascular dysfunction, pulmonary hypertension (PH), and resulting right ventricular (RV) failure occur in many critical illnesses and may be associated with a worse prognosis. PH and RV failure may be difficult to manage: principles include maintenance of appropriate RV preload, augmentation of RV function, and reduction of RV afterload by lowering pulmonary vascular resistance (PVR). We therefore provide a detailed update on the management of PH and RV failure in adult critical care.

**Methods:**

A systematic review was performed, based on a search of the literature from 1980 to 2010, by using prespecified search terms. Relevant studies were subjected to analysis based on the GRADE method.

**Results:**

Clinical studies of intensive care management of pulmonary vascular dysfunction were identified, describing volume therapy, vasopressors, sympathetic inotropes, inodilators, levosimendan, pulmonary vasodilators, and mechanical devices. The following GRADE recommendations (evidence level) are made in patients with pulmonary vascular dysfunction: 1) A weak recommendation (very-low-quality evidence) is made that close monitoring of the RV is advised as volume loading may worsen RV performance; 2) A weak recommendation (low-quality evidence) is made that low-dose norepinephrine is an effective pressor in these patients; and that 3) low-dose vasopressin may be useful to manage patients with resistant vasodilatory shock. 4) A weak recommendation (low-moderate quality evidence) is made that low-dose dobutamine improves RV function in pulmonary vascular dysfunction. 5) A strong recommendation (moderate-quality evidence) is made that phosphodiesterase type III inhibitors reduce PVR and improve RV function, although hypotension is frequent. 6) A weak recommendation (low-quality evidence) is made that levosimendan may be useful for short-term improvements in RV performance. 7) A strong recommendation (moderate-quality evidence) is made that pulmonary vasodilators reduce PVR and improve RV function, notably in pulmonary vascular dysfunction after cardiac surgery, and that the side-effect profile is reduced by using inhaled rather than systemic agents. 8) A weak recommendation (very-low-quality evidence) is made that mechanical therapies may be useful rescue therapies in some settings of pulmonary vascular dysfunction awaiting definitive therapy.

**Conclusions:**

This systematic review highlights that although some recommendations can be made to guide the critical care management of pulmonary vascular and right ventricular dysfunction, within the limitations of this review and the GRADE methodology, the quality of the evidence base is generally low, and further high-quality research is needed.

## Introduction

Pulmonary vascular dysfunction is a broad term and may be central to several disease processes in the intensive care unit (ICU). Components include pulmonary endothelial dysfunction, altered lung microvascular permeability, vasoactive mediator imbalance, abnormal hypoxic vasoconstriction, pulmonary metabolic failure, microvascular thrombosis, and later, vascular remodelling [1-3]. The resulting elevation in pulmonary vascular resistance (PVR) and pulmonary hypertension (PH) may increase the transpulmonary gradient, and the right ventricular "pressure overload" can in turn result in right ventricular (RV) dysfunction and failure [4]. RV dysfunction may also result from volume overload or a primary RV pathology reducing contractility, including RV infarction and sepsis (Table 1) [4-7].

**Table 1:** Causes of pulmonary hypertension and right ventricle failure in the ICU

Causes of pulmonary hypertension in ICU	Causes of RV failure in ICU
1) PAH (for example, preexisting PAH; PoPH (8.5% ESLD)	1) RV Pressure overload, pulmonary hypertension, any cause
2) Elevated LAP: RV pressure overload (left-sided myocardial infarction/cardiomyopathy; mitral regurgitation; pulmonary stenosis)	2) Reduced RV contractility
3) PH due to hypoxia: acute (for example, ARDS)/preexisting lung disease (for example, COPD, IPF)	RV infarction; sepsis; RV cardiomyopathy; myocarditis; pericardial disease; LVAD; after CPB; after cardiac surgery/transplantation
4) Thromboembolic (for example, acute PE; chronic (CTEPH); other causes of emboli (AFE, air, cement)	3) RV-volume overload
5) Mechanical (for example, increased Pplat - IPPV	Cardiac causes: tricuspid and pulmonary regurgitation; intracardiac shunts

AFE, amniotic fluid embolus; ARDS, acute respiratory distress syndrome; COPD, chronic obstructive pulmonary disease; CPB, cardiopulmonary bypass; CTEPH, chronic thromboembolic pulmonary hypertension; ESLD, end-stage liver disease; IPF, idiopathic pulmonary fibrosis; IPPV, intermittent positive-pressure ventilation; LAP, left atrial pressure; LVAD, left ventricular assist device; PAH, pulmonary arterial hypertension; PoPH, portopulmonary hypertension; P_plat_, plateau pressure; RV, right ventricle.

PH is defined at right-heart catheterization in the outpatient setting, with resting mPAP exceeding 25 mm Hg, and a PVR greater than 240 dyn.s.cm^-5 ^(3 Wood units) [8]. At echocardiography, the presence of PH is suggested by the estimated RV systolic pressure (RVSP) exceeding 35 mm Hg (being severe if >50 mm Hg) (see later) [9], and the pulmonary arterial acceleration time (PAT) may be shortened [10]. Pulmonary arterial hypertension (PAH) defines PH not due to left-heart disease, with PAOP <15 mm Hg or without echocardiographic evidence of increased left atrial pressure. The severity of PH may depend on the chronicity: the actual pulmonary artery pressure generated will increase with time as the RV hypertrophies.

RV dysfunction describes reduced RV contractility, which may be detected in several ways. At echocardiography, RV distention causes the intraventricular septum to deviate, with resulting paradoxic septal movement that impinges on LV function [11]. RV function may be difficult to assess on echocardiography, especially in ventilated patients, and measurement of the descent of the RV base toward the apex (tricuspid annular systolic excursion, TAPSE) or RV fractional shortening may useful [12,13]. Invasive monitoring may show a CVP exceeding the PAOP, or increasing CVP and PVR with a decreasing cardiac output (and mPAP may therefore decrease), and high right ventricular end-diastolic filling pressure is characteristic. By using an RV ejection fraction (RVEF) PAC, an increase in RV end-diastolic index and a reduction in RVEF are seen [14]. We have defined RV failure to be the clinical result of RV dysfunction with the onset of hypotension or any resulting end-organ (for example, renal, liver, or gastrointestinal) dysfunction. Acute cor pulmonale (ACP) refers to acute right heart failure in the setting of acutely elevated PVR due to pulmonary disease [15,16].

Pulmonary hypertension *per se *is frequently encountered in the ICU. It is commonly due to elevated pulmonary venous pressure in the setting of left-sided heart disease, or in patients with preexisting pulmonary vascular disease. It is well recognized after cardiothoracic surgery, in part related to the endothelial dysfunction seen with cardiopulmonary bypass (CPB) [17,18]. PH is also associated with sepsis [19]; acute respiratory distress syndrome (ARDS) [20-22] (with associated acute RV failure in 10% to 25% of cases [23,24]), and in up to 60% of patients after massive pulmonary embolism (PE) [25]. PH is important to recognize in the ICU because its presence predicts increased mortality in these conditions [19,23,25-31] as well as after surgical procedures [32-42]. Mortality from cardiogenic shock due to RV infarction (> 50%) exceeds that due to LV disease [5]. We therefore thought that a systematic review of the current evidence for the management of PH, resulting RV dysfunction, and failure in adult patients in the ICU, would be a useful addition to the critical care literature.

### The pulmonary circulation and pathophysiology of right ventricular failure

**Figure 1:**
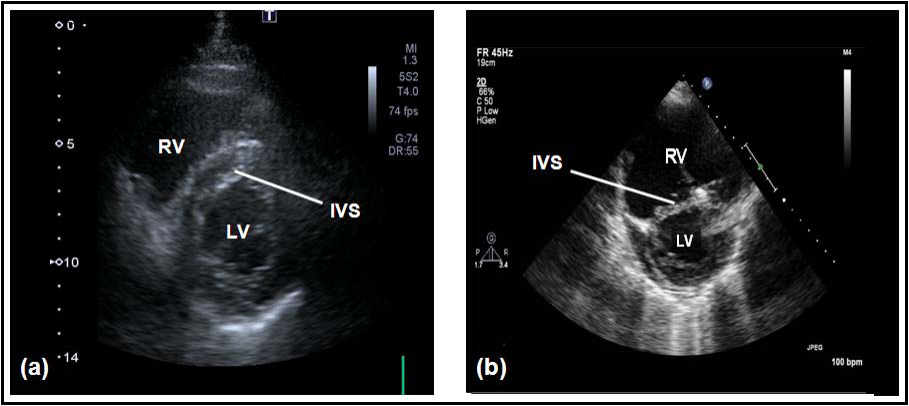
**Short-axis view of a transthoracic echocardiogram in a normal subject (a) and a patient with an acutely dilated right ventricle (RV) in the setting of high pulmonary vascular resistance (b)**. The intraventricular septum (IVS) is D-shaped in (b), reflecting the acute RV pressure overload in this patient, and marked enlargement of the RV in (b) compared with (a). Courtesy of Dr Susanna Price, Royal Brompton Hospital, London, UK.

The normal pulmonary circulation is a high-flow, low-pressure system. Unlike the left ventricle (LV), the thin-walled right ventricle tolerates poorly acute increases in afterload. This may lead to acute distention (Figure 1) [4,43], with a resulting increase in oxygen consumption and reduction in contractility [44]. The dilated RV, together with paradoxic intraventricular septal movement [45], lead to reduced LV filling [46], cardiac output (CO), and oxygen delivery [47]. The principle of ventricular interdependence is important in most settings: superficial myocardial fibers encircle both ventricles; thus they are contained within the same pericardial cavity (except maybe after cardiac surgery), as well as sharing a septum, effectively existing "in series" [48,49]. This explains the decrease in LV output seen during positive-pressure ventilation [48,50,51] and why RV pressure and volume overload cause diastolic dysfunction of the LV [52]. Furthermore, because of the RV/LV interactions, the LV may markedly depend on atrial contraction for filling and may tolerate atrial fibrillation and vasodilating therapy particularly poorly [49,53,54].

**Figure 2:**
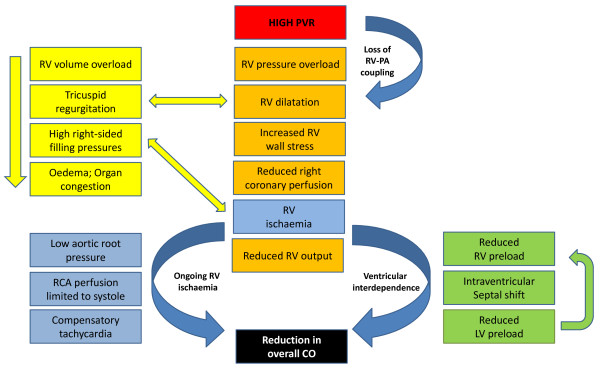
**Pathophysiology of right ventricular failure in the setting of high PVR**. CO, cardiac output; LV, left ventricle; MAP, mean arterial pressure; PVR, pulmonary vascular resistance; RV, right ventricle.

In addition, perfusion of the right coronary artery is usually dependent on a pressure gradient between the aorta and the right ventricle, which, in the setting of increased RV afterload and decreased coronary blood flow, may lead to RV ischemia [55], with further severe hemodynamic decompensation [56] (Figure 2). In acute-on-chronic RV-pressure overload, the already-hypertrophied RV tolerates much higher pressures before decompensation [57,58], although the ability of the RV to augment CO in chronic PH may be restricted by its relatively "fixed" afterload. In any setting, the most common cause of increased RV afterload is an increase in PVR (Table 2).

**Table 2:** Local factors increasing pulmonary vascular tone

Factors increasing pulmonary vascular tone	Additional contributors to elevated PVR in ARDS
High pulmonary arterial pCO_2_/low pH	Vasoconstrictor: vasodilator imbalance
Low mixed venous pO_2_	Excess ET-1 [361], TXA-1, PDE, 5HT [2]
High sympathetic tone; α-adrenoceptor agonism	Reduced NO, prostanoids [20]
Mechanical effects:	Effects of endotoxin [22,362]
High airway P_plat_; gravity; increased flow (for example, one-lung ventilation)	Endothelial injury [363]
Relating to CPB:	Hypoxic vasoconstriction (80% arteriolar) [22,364]
Preexisting PH; endothelial injury [17]; protamine [18]	Microthrombosis, macrothrombosis [62,365]
	Pulmonary vascular remodeling [1]

5-HT, serotonin; ARDS, acute respiratory distress syndrome; CPB, cardiopulmonary bypass; ET-1, endothelin-1; NO, nitric oxide; pCO_2_, partial pressure of carbon dioxide; PDE, phosphodiesterase; pO_2_, partial pressure of oxygen; P_plat_, plateau pressure; PVR, pulmonary vascular resistance; TXA-1, thromboxane A1.

**Figure 3:**
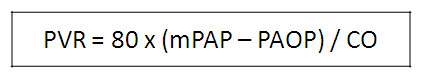
**Calculation of pulmonary vascular resistance**. Normal range, 155-255 dynes/sec/cm^5^. CO, cardiac output; mPAP, mean pulmonary artery pressure; PAOP, pulmonary arterial occlusion pressure.

The gold standard for the diagnosis and management of PH and RV dysfunction in the ICU setting is considered by some to be through pulmonary artery catheterization (PAC), even though most of the information can be obtained noninvasively by echocardiography: the requirement for PAC in this population remains controversial. It must, however, be acknowledged that it provides the only direct continuous measurement of right-sided pressures and direct measurement of RV afterload, whereby, through measurement of cardiac output, pulmonary pressures and the pulmonary artery occlusion pressure (PAOP, the "wedge"), the PVR can be calculated (Figure 3). Overall outcomes are not improved when the PAC is used in general in critically ill patients; and complications do occur [59]: the use in general is therefore declining. However, no studies have been done in the "pulmonary vascular" subpopulation. Alternative invasive hemodynamic measurements, such as CVP, may be useful surrogates for volume status in RV failure, by using the diastolic component of the CVP. Importantly, when monitoring CVP in patients with significant tricuspid regurgitation (TR), the variable V wave may be misleading, as it is included in the mean CVP calculation on most automated machines, and if rising, indicates RV overdistention. In the setting of cardiac surgery, one study shows that PAC use has reduced from 100% to 9% from 1997 to 2001, thought to reflect increased use of transesophageal echocardiography (TEE) [60]. In the setting of cardiac surgery, PAC may remain indicated for patients with PH and low CO and those predicted to have a difficult postoperative course [60], when a Swan introducer sheath may be inserted preemptively, or inserted for continuous monitoring after a diagnosis of RV dysfunction made with echocardiography [61]. PAC is also a useful cardiac monitor with intraaortic balloon counterpulsation. Few data exist on PAC in other settings of pulmonary vascular dysfunction in the ICU, but one study suggests that PVR may be a poor indicator of pulmonary-circulation status in ventilated patients with ALI/ARDS [62]. The role of echocardiography, both transthoracic (TTE) and TEE, is increasingly recognized in assessing RV function in many ICU settings [63-65] and provides essential information about RV geometry and function. PA pressures may be assessed by estimating the systolic-pressure gradient across the tricuspid valve by using the modified Bernoulli equation [9,66,67], and although the correlation between invasive and sonographic measurement has been shown to be excellent in these studies, no studies have correlated PAC with echocardiographic measurements in the ICU population. In reality, a combination of invasive and noninvasive techniques is used. Biomarkers such as brain natriuretic peptide (BNP) are useful in monitoring chronic PAH [68], in risk-stratifying acute pulmonary embolism (see later) [69-71], and in identifying ARDS-related pulmonary vascular dysfunction [72], although their role is less clear in other ICU settings.

The diagnosis and management of acute pulmonary embolism (PE) warrants a specific mention, as it is a relatively common cause of acute RV failure in the ICU [73]. Available therapies include thrombolysis and embolectomy, reducing the clot burden and acute mortality [74,75], as well as reducing the longer-term risk of chronic thromboembolic PH [76]. Given that more than half of related deaths occur within an hour of the onset of symptoms [77], effective supportive treatment of shock is paramount. Patients presenting with acute PE are risk stratified according to the effects of elevated RV afterload: hypotensive patients and those with elevated cardiac biomarkers or echocardiographic indices of RV strain, or both, are deemed at increased risk, and thrombolysis is indicated [78].

The management of PH and RV dysfunction in the ICU is challenging. No agreed algorithms exist, although treatment should aim to prevent pulmonary hypertensive crises and acute cor pulmonale [79]. These comprise the spectrum of acute pulmonary vascular dysfunction and may result in cardiovascular collapse due to resulting biventricular failure. Management principles include the following: 1) optimization of RV preload, 2) optimization of RV systolic function, 3) reduction of afterload by reduction of increased PVR, and 4) maintenance of aortic root pressure to ensure sufficient right coronary artery filling pressure (Table 3).

**Table 3:** Management principles in pulmonary vascular dysfunction

1. Optimize volume status: avoid filling (± offload) if RV volume-overloaded
2. Augment CO
3. Reduce PVR
a) Use pulmonary vasodilators (preferably inhaled: less systemic hypotension and V/Q mismatch)
b) Treat reversible factors that may increase PVR
Metabolic state: correct anemia, acidosis, hypoxemia
Treat respiratory failure: treat hypoxia; limit P_plat _by using lung-protective ventilatory strategies, but beware of high pCO_2 _increasing PVR
Reduce sympathetic overstimulation
4. Maintain adequate systemic vascular resistance (SVR): keep PVR well below SVR; use pressors if necessary

## Materials and methods

### Systematic review of ICU management of pulmonary vascular and RV dysfunction

We performed a systematic review of the literature over the period from 1980 to 2010, by using set search terms, and the electronic database of the US National Library of Medicine and National Institute of Health (PubMed). After initial identification, abstracts were reviewed for relevance, and appropriate studies were included in the review. Reference lists of relevant articles were hand-searched for further studies and reports. The search was limited to publications in English. Studies were deemed suitable for inclusion according to the criteria listed and where the patient population and study design was defined; and the outcomes were limited to those depending on the specific GRADE question (see Additional file 1). The breakdown of articles obtained by the systematic search is shown (Table 4). After identification, relevant studies were included and subjected to a GRADE analysis [80,81] to see whether we could make specific management recommendations.

**Table 4:** Breakdown of clinical articles

Subtype of treatment for pulmonary vascular dysfunction	Number of studies in initial search	Number of suitable studies included in review
Volume therapy	113	5
Vasopressors	388	28
Sympathetic inotropes	565	8
Inodilators	280	17
Levosimendan	172	12
Pulmonary vasodilators	586	121
Mechanical devices	47	19

## Results and Discussion

### ICU management of pulmonary vascular and RV dysfunction

Management of PH with associated RV dysfunction in the ICU setting can be broken down into several treatment goals (Table 3). The first is to ensure adequate but not excessive RV filling or preload in the context of sufficient systemic blood pressure. The second goal is to maximize RV myocardial function, whether with inotropic support, rate or rhythm management, atrioventricular synchronization [82,83], or by using mechanical devices. The third is to offload the right ventricle by reducing the PVR with pulmonary vasodilators as well as by ensuring adequate oxygenation, avoiding hypercapnia and acidosis, and by minimizing mechanical compression of pulmonary vessels (for example, due to excessive airway plateau pressure). The fourth is to maintain adequate aortic root pressure to allow sufficient right coronary arterial perfusion.

### Management of volume and use of vasopressors

Systemic hypotension may relate to sepsis, overdiuresis, or progression of RV failure itself. Principles of volume management and vasopressor use are summarized.

#### Volume management

With a normal RV, RV ejection fraction is usually primarily dependent on RV preload [84]. In the setting of excessive myocardial distention (by fluids), wall tension increases according to the Frank-Starling mechanism, and muscle fiber length is increased, beyond a certain point at which ventricular function will fail. This situation may be precipitated sooner in the setting of PH and RV dysfunction, in which both hypo- and hypervolemia may reduce cardiac output [78,85,86]. In stable patients with PAH, high plasma volumes are associated with worse outcomes [87], but very few clinical studies have been performed in pulmonary vascular dysfunction, and the use of fluid loading remains controversial. Some animal studies show that fluids increase the cardiac index [88]; others show that they worsen shock by inducing RV ischemia or decreasing LV filling or both as the result of ventricular diastolic interdependence (due to an increase in RV volume) [89-91].

In acute cor pulmonale after massive PE, increased filling may be at least initially required [4,92]. In observational studies in sepsis, up to 40% of patients have evidence of RV failure [93], predominantly due to primary RV dysfunction [7]. These patients have a higher CVP at baseline [94] and are unable to augment stroke volume or perfusion pressure with fluid challenges alone, and so usually also require catecholamines [93,94].

RV volume overload is a very important principle to recognize and treat promptly in RV failure. It may be identified by a rising V wave on the CVP trace, or by increased TR due to RV overdistention seen at echocardiography. In this situation of "backwards" heart failure, no further escalation of vasoactive agents is likely to be helpful (and may even be harmful), and management involves fluid removal (by using diuresis [95] or hemofiltration [96]) and avoidance of excessive RV afterload [97]. Unmonitored fluid challenges are inadvisable in any setting of RV failure [98,99].

### GRADE RECOMMENDATION 1

Based on overall very-low-quality evidence (see Additional file 1), the following WEAK recommendation is made: Close monitoring of fluid status according to effects on RV function is recommended. Initial carefully monitored limited volume loading may be useful after acute PE, but may also worsen RV performance in some patients with pulmonary vascular dysfunction, and vasoactive agents may be required.

#### Vasopressors

An essential goal is to maintain systemic blood pressure above pulmonary arterial pressures, thereby preserving right coronary blood flow: unlike left coronary artery perfusion, which occurs only during diastole (as aortic pressure exceeds LV pressure only during this period), perfusion of the right coronary artery usually occurs throughout the cardiac cycle, dominating in systole. It is understood that, as PVR approaches SVR, coronary perfusion will decrease, and if PVR exceeds SVR, coronary filling will occur only in diastole. By augmenting aortic root pressure by using vasopressors in the setting of increased RV afterload, RV ischemia can therefore be reversed [55]. Vasopressors will, however, inevitably have direct effects on the pulmonary circulation as well as myocardial effects (Table 5).

**Table 5:** Pulmonary vascular properties of vasoactive agents

	CI	PVR	SVR	PVR/SVR	Tachycardia	**Renal**^**a**^**/metabolic**
Vasopressors				Dose related		
NE	+	+	++	+/-	+	Lactic acidosis
PHE	-	++	+	+	-	-
Low-dose AVP	+/-	+/-	++	-	-	Diuresis ++
Inotropes						
Dobutamine	++	-	-	-	+	
< 5 μg/kg/min						
Dopamine	+	+/-	+	+	++	Natriuresis
Epinephrine	++	-	++	-	++	Lactic acidosis
Inodilators						
PDE IIIs	++	-	-	-	+/-	-
Levosimendan	++	-	-	-	-	-

AVP, arginine vasopressin; NE, norepinephrine; PDE IIIs, phosphodiesterase inhibitors; PHE, phenylephrine. **^a^**Renal blood flow is likely to improve with increased cardiac output and systemic blood pressure with all agents.

##### Sympathomimetic pressors

These include the catecholaminergic pressor, norepinephrine, and the noncatecholaminergic pressor phenylephrine. Their complex effects on the pulmonary circulation depend on the dose-related relative α- and β-adrenoreceptor stimulation as well as the degree and nature of RV dysfunction [99,100]. All may potentially lead to tachydysrhythmias, diastolic dysfunction, myocardial ischemia, hyperlactatemia, and hypercoagulability [101].

###### Norepinephrine

Norepinephrine (NE) exerts its systemic vasopressor effects through α-1 agonism [102]. Activation of these receptors also causes pulmonary vasoconstriction [102,103], although the potential adverse effects on PVR are likely to occur only at high doses. Most evidence supporting this comes from animal studies in models of pulmonary vascular dysfunction, with NE at doses less than 0.5 μg/kg/min not increasing PVR [44]. In persistent PH of the newborn, low-dose NE (0.5 μg/kg/min) reduces the PVR/SVR ratio [104]. In adults with septic shock, higher doses of NE increase PVR/SVR, although without worsening RV performance [105]. In patients with sepsis, PH, and associated RV dysfunction, NE increases SVR and improves the RV oxygen supply/demand ratio, although it does not increase RVEF and does increase PVR [106]. Importantly, NE is positively inotropic through β-1 receptor agonism, thus improving RV/pulmonary arterial coupling, CO, and RV performance in studies of acute RV dysfunction due to PH [44,89,107-109], illustrated in a case report of acute PH after MVR surgery [110]. In patients with chronic PH, NE reduces the PVR/SVR ratio, although it may not improve CI [100], which may relate to the "fixed" elevation in PVR [99].

###### Phenylephrine

Phenylephrine (PHE) is a direct α-agonist. Its use improves right coronary perfusion in RV failure [55] without causing tachycardia, although this benefit may be offset by worsening RV function due to increased PVR [100,108,111].

### GRADE RECOMMENDATION 2

Based on mostly low-quality evidence (see Additional file 1), the following WEAK recommendation is made: NE may be an effective systemic pressor in patients with acute RV dysfunction and RV failure, as it improves RV function both by improving SVR and by increasing CO, despite potential increases in PVR at higher doses.

#### Nonsympathomimetic pressors: Vasopressin

Arginine *v*asopressin (AVP) causes systemic vasoconstriction via the vasopressinergic (V1) receptor. Experimental studies have revealed vasodilating properties at low doses that include pulmonary vasodilatation [112] through an NO-dependent mechanism via V_1 _receptors [113,114]. This property manifests clinically as a reduction in PVR and PVR/SVR ratio [105,115,116]. AVP has also been used as a rescue therapy in patients during PH crises [117-119], in which untreated equalization of systemic and pulmonary pressures may be rapidly fatal. At low doses (0.03-0.067 U/min), it has been used safely in sepsis [105,120-124], as well as in patients with acute PH and RV failure with hypotension after cardiac surgery [115,116,125,126] and hypotension associated with chronic PH in several settings [117,118,127,128].

AVP leads to a diuretic effect in vasodilatory shock [129], reduces the heart rate [105,121,130-132], and induces fewer tachyarrhythmias in comparison to NE [105,131]. However, bradycardia [133] may be encountered at high clinical doses [134,135]. AVP may cause dose-related adverse myocardial effects at infusion rates exceeding 0.4 U/min [134,135], or even above 0.08 U/min in cardiogenic shock [136], which probably relate to direct myocardial effects, including coronary vasoconstriction [132,137-139].

### GRADE RECOMMENDATION 3

Based on mostly low-quality evidence (see Additional file 1), the following WEAK recommendation is made: In patients with vasodilatory shock and pulmonary vascular dysfunction, low-dose AVP may be useful in difficult cases that are resistant to usual treatments, including norepinephrine.

### Inotropic augmentation of RV myocardial function

The next major goal is to improve RV myocardial function by using inotropes. The use of mechanical support is discussed later. For sympathomimetic agents, desirable cardiac β_1 _effects at lower doses maybe offset by chronotropic effects precipitating tachyarrhythmias [140], as well as worsening pulmonary vasoconstriction at higher doses [102] through α-agonism. Systemic hypotension may result from these agents and with phosphodiesterase inhibitors, which may necessitate co-administration of vasopressors.

#### Inotropes

##### Sympathomimetic inotropes

Few clinical studies of these agents have been done in patients with PH and RV dysfunction. Dopamine increases CO, although it may cause a mild tachycardia in patients with PH [141] and increase the PVR/SVR ratio [142]. Dopamine also tends to increase the heart rate and to have less-favorable hemodynamic effects in patients with cardiomyopathy than dobutamine [143], although it does not increase PVR at doses up to 10 μg/kg/min in animals with pulmonary vascular dysfunction [144]. In patients with septic shock, PH, and RV dysfunction, dopamine improves CI without an increase in PVR [145]. In the recent large randomized controlled study comparing dopamine with norepinephrine in patients with septic shock, dopamine increased arrhythmic events and, in patients with cardiogenic shock, increased the risk of death [146]. In patients with primary RV dysfunction (without PH) due to septic shock, epinephrine improves RV contractility despite an 11% increase in mPAP [14]. In animal studies, epinephrine reduces the PVR/SVR more than does dopamine [147]. Isoproterenol has been used in RV failure primarily as a chronotrope after cardiac transplantation [148], although it may induce arrhythmias [149].

###### Dobutamine

At clinical doses up to 5 μg/kg/min in heart failure, dobutamine increases myocardial contractility, reduces PVR and SVR, and induces less tachycardia than does dopamine [143]. It improves RV performance in patients with PH at liver transplantation [150], after RV infarction[151], and is used in PAH exacerbations [152]. It is synergistic with NO in patients with PH [153]. Experimentally, dobutamine has favorable pulmonary vascular effects at lower doses [44,154], although it leads to increased PVR, tachycardia, and systemic hypotension at doses exceeding 10 μg/kg/min [155]. Given the adverse effects of systemic hypotension in these patients, it is important to anticipate and treat it with vasopressors when using dobutamine.

##### Inodilators

An inodilator increases myocardial contractility while simultaneously causing systemic and pulmonary vasodilatation. Inodilators include the phosphodiesterase (PDE) III inhibitors and levosimendan.

###### PDE3 inhibitors

Several types of PDE are recognized: PDEIII usually deactivates intracellular cyclic adenosine monophosphate (cAMP), and PDE3 inhibitors therefore increase cAMP and augment myocardial contractility while dilating the vasculature [156-158]. The selective PDEIII inhibitors include enoximone, milrinone, and amrinone. They are most suited to short-term use because of tachyphylaxis [159], and mild tachycardia is common. Milrinone is most frequently used and has been shown to reduce pulmonary pressures and augment RV function in many studies in patients with pulmonary vascular dysfunction [160-164]. Enoximone improves RV function in pulmonary vascular dysfunction after cardiac surgery [165,166] and in patients with decompensated chronic obstructive pulmonary disease (COPD) [167]. Enoximone leads to fewer postoperative myocardial infarctions than does dobutamine [168,169], which may relate to the resulting improved gas exchange when compared with dobutamine and GTN [170]. Concerns regarding platelet aggregation with amrinone [171] do not appear to arise with enoximone [172] or milrinone after cardiac surgery [173,174]. As with dobutamine, resulting reversible systemic hypotension means that coadministration with pressors is often necessary. Agents such as norepinephrine, phenylephrine or vasopressin are used, with the latter reducing PVR/SVR more than norepinephrine [115]. PDEIII inhibitors may also improve RV function in chronic PH [175].

Nebulized milrinone is increasingly used to manage PH crises in several settings [176-179]. Through pulmonary selectivity, it results in less systemic hypotension and less V/Q mismatch compared with intravenous use in patients with PH after mitral valve replacement surgery [177,178]. The combination of milrinone-AVP reduces PVR/SVR and may be preferable to milrinone-NE in RV dysfunction [115].

###### Levosimendan

Levosimendan sensitizes troponin-C to calcium and selectively inhibits PDE III, improving diastolic function and myocardial contractility without increasing oxygen consumption [180-183]. It also acts as a vasodilator through calcium desensitization, potassium channel opening, and PDEIII inhibition [184]. Levosimendan leads to a rapid improvement in hemodynamics, including reduction in PVR in patients with decompensated heart failure [185], with significant benefit on RV efficiency [182], with effects lasting several days [186]. Levosimendan improves RV-PA coupling in experimental acute RV failure [187-189] more than dobutamine [188]. These effects have been shown clinically with improvements in RV function and reduction in PVR in ischemic RV failure [190-194], ARDS [195], and after mitral valve replacement surgery [196,197]. In chronic PH, repetitive doses reduce mPAP and PVR from baseline and improve SvO_2 _[198].

### GRADE RECOMMENDATION 4

Based on low-moderate-quality evidence (see Additional file 1), a WEAK recommendation can be made that low-dose dobutamine (up to 10 μg/kg/min) improves RV function and may be useful in patients with pulmonary vascular dysfunction, although it may reduce SVR. Dopamine may increase tachyarrhythmias and is not recommended in the setting of cardiogenic shock (STRONG recommendation based on high-quality evidence level).

### GRADE RECOMMENDATION 5

Based on mostly moderate-quality evidence (see Additional file 1), a STRONG recommendation can be made that PDE III inhibitors improve RV performance and reduce PVR in patients with acute pulmonary vascular dysfunction, although systemic hypotension is common, usually requiring coadmininstration of pressors. Based on low-quality evidence (see Additional file 1), a WEAK recommendation can be made that inhaled milrinone may be useful to minimize systemic hypotension and V/Q mismatch in pulmonary vascular dysfunction.

### GRADE RECOMMENDATION 6

Based on mostly low-quality evidence (see Additional file 1), a WEAK recommendation can be made that levosimendan may be considered for short-term improvements in RV performance in patients with biventricular heart failure.

### Reduction of right ventricular afterload

Physiologic coupling between the RV and the pulmonary circulation is a vital form of autoregulation of pulmonary circulatory flow (Figure 2). The RV is even less tolerant of acute changes in afterload than the LV, presumably because of the lower myocardial muscle mass [199]. In sepsis, a reduction in PVR will increase the RV ejection fraction at no additional cost to cardiac output [47], but at levels beyond moderate PH, LV filling may be reduced, and ultimately cardiac output will decrease [199]. Measures to reduce RV afterload may be nonpharmacologic (Table 3) or pharmacologic (Table 6).

**Table 6:** Agents used to reduce PVR in the ICU setting

Drug	Dose	Half-life (duration of action)	Potential adverse effects
Intravenous			
Prostacyclin (Epoprostenol, Flolan)	Start at 1 ng/kg/min; titrate upward in 2-ng/kg/min increments according to effect	3-5 minutes (10 minutes)	Systemic hypotension, worsening oxygenation (increased V/Q mismatch), antiplatelet effect, headache, flushing, jaw pain, nausea, diarrhea
Iloprost	1-5 ng/kg/min	30 minutes	Similar to Flolan; also syncope (5%)
Sildenafil [325] (NB off-license use in hemodynamically unstable patients)	Low dose, 0.05 mg/kg; high dose, 0.43 mg/kg) (comes as 0.8 mg/ml)	3-5 hours	Hypotension: caution if fluid depleted, severe LV-outflow obstruction, autonomic dysfunction. Hypoxemia due to V/Q mismatch. Common: headache, flushing, diarrhea, epistaxis, tremor. Rare but important: anterior ischemic optic neuropathy
Milrinone	50 μg/kg over 10 minutes followed by 0.375-0.75 μg/kg/min infusion	1-2 hours	Tachyarrhythmias, hypotension
Adenosine	50-350 μg/kg/min, titrate up in 50 μg/kg/min increments	5-10 seconds (2 minutes)	Bradycardia, bronchospasm, chest pain
Inhaled (preferred; Note variable absorption likely)			
Prostacyclin (Epoprostenol, Flolan) [286,303]	0.2-0.3 ml/min of 10-20 μg/ml nebulized into inspiratory limb of ventilator circuit (30-40 ng/kg/min)	3-5 minutes	As above but less hypotension and improved oxygenation compared with intravenous use
Iloprost [275]	2.5-5 μg 6-9 times/day, 1 mg/ml milrinone into the ventilator circuit at 0.2-0.3 ml/min for 10-20 minutes	30 minutes	As above and bronchospasm
Milrinone [176,178,179]	5-80 ppm continuously	1-2 hours	Less systemic hypotension than with IV milrinone
NO		15-30 seconds (5 minutes)	Methemoglobinemia; withdrawal PH
ORAL (rarely in ICU)			
Bosentan	62.5-125 mg b.d.	5 hours	Liver-function test abnormalities; drug interactions; edema
Sildenafil	0.25-0.75 mg/kg 4 hrly	3-4 hours	As above; less hypotension and hypoxemia in stable patients

#### Pulmonary vasodilator therapy

Specific pulmonary vasodilators may be useful both to reduce RV afterload and to manipulate hypoxic vasoconstriction in patients with severe hypoxia. Agents are classically subdivided according to their action on the cyclic GMP, prostacyclin, or endothelin pathways [200]. In the nonacute setting, these agents also target remodeling of"resistance" pulmonary vessels and have revolutionized the care of patients with PAH [201]. Importantly, however, the management with pulmonary vasodilators in chronic PH patients differs in several ways from that with acute pulmonary vascular dysfunction, notably in terms of rapid changes in RV volume status, and potential adverse hemodynamic effects of nonselective pulmonary vasodilators in unstable patients.

Pulmonary vasodilators should be used after optimization of RV perfusion and CO. Systemic administration of pulmonary vasodilators may reduce systemic blood pressure [202], potentially reducing RV preload and worsening RV ischemia [86]. Exclusion of a fixed elevated pulmonary venous pressure is important, as increased transpulmonary flow may precipitate pulmonary edema [203,204]. Furthermore, nonselective actions of vasodilators may result in worsening ventilation/perfusion (V/Q) matching [205]. This risk is reduced with the use of inhaled pulmonary vasodilators, with which the agent will reach vessels in only ventilated lung units [206].

##### Adenosine

Adenosine increases intracellular cAMP via A_2 _receptor agonism [207], and when administered intravenously, acts as a potent selective pulmonary vasodilator because of its rapid endothelial metabolism [208]. It has been used as a therapy for adult PH in some settings, including after cardiac surgery [209], but may elevate LV end-diastolic pressure [210] and cause bradycardia and bronchospasm [211]. It is currently therefore recommended as an alternative to NO and prostacyclin in dynamic vasoreactivity studies rather than as treatment for PH [201].

##### Inhaled nitric oxide

Inhaled nitric oxide (NO) is a potent pulmonary vasodilator with a short half-life due to rapid inactivation by hemoglobin. This minimizes systemic vasodilatation, although it necessitates continuous delivery into the ventilator circuit [206]. NO selectively reduces PVR and improves CO in PAH [212], secondary PH [205,213,214], acute PE [215,216], ischemic RV dysfunction [217,218], and postsurgical PH [202,219-234]. NO also improves oxygenation [235], RVEF, and reduces vasopressor requirements in PH after cardiac surgery [236], especially in patients with higher baseline PVR [237], with no augmented effect seen at doses above 10 ppm in these patients [238]. Use of NO (or inhaled PGI_2_) after mitral valve replacement surgery results in easier weaning from cardiopulmonary bypass and shorter ICU stays [239,240].

NO has been shown to reduce PVR and improve CO in several studies in patients with acute RV failure due to ARDS [79,241-246] and to improve oxygenation at lower doses than the RV effects [247]. Administration of NO does need to be continuous for PVR reduction, and a potential exists for worsening oxygenation at excessive doses [248]. The reduction in RV afterload, however, does not correlate with clinical-outcome benefits [249-251]. Similarly, despite short-term improvements in oxygenation in ARDS [252], no studies show a survival benefit [249,250,253-257].

NO provides synergistic pulmonary vasodilatation with intravenous prostacyclin [258], inhaled iloprost [259], and oral sildenafil [260,261]. Limitations include accumulation of toxic metabolites, although this is not usually a clinically significant problem [206]. Rebound PH with RV dysfunction may occur after weaning from NO [262-264], which may be reduced with PDE5 inhibitors [265-270].

##### Prostanoids

Prostanoids include prostaglandin-I_2 _(prostacyclin, PGI_2_) and its analogues, (iloprost) and prostaglandin-E_1 _(alprostadil, PGE_1_). An important difference between their formulations is their resulting half-life (Table 6). Prostacyclin is a potent systemic and pulmonary vasodilator, with antiplatelet [271] and antiproliferative effects [272]. In PAH, these agents reduce PVR, increase CO, and improve clinical outcomes [273-279], and are used in patients with NYHA III-IV symptoms [201].

The use of prostanoids is most commonly described in ICU after cardiac surgery or transplantation. Intravenous prostacyclin [18,280], PGE_1 _[281-285], inhaled prostacyclin [223,286-290], and iloprost [291-297] all reduce PVR and improve RV performance in these settings, with inhaled agents being most selective. Intravenous PGE_1 _may cause marked desaturation in patients with lung disease [205]. Inhaled prostacyclin has short-term equivalence to NO [226], and inhaled iloprost has been shown to be even more effective than NO at acutely reducing PVR and augmenting CO in PH after CPB [298] and in PAH [277]. Inhaled PGI_2 _also acutely improves pulmonary hemodynamics after acute massive PE [299]. Although PGI_2 _impairs platelet aggregation, clinical bleeding was not increased in one study [300]. The potential anticoagulant effect should be remembered, however, especially in patients after surgery and receiving concomitant heparin.

In ARDS, intravenous prostacyclin reduces PVR and improves RV function, although it may increase intrapulmonary shunt [301]. Inhaled prostacyclin [302-305] and inhaled PGE_1 _[306] improve oxygenation and reduce PVR in ARDS, with minimal effects on SVR. NO and intravenous PGI_2 _have been combined in ARDS with effective reduction of PVR without adverse effects [307].

##### PDE5 inhibitors

**Figure 4:**
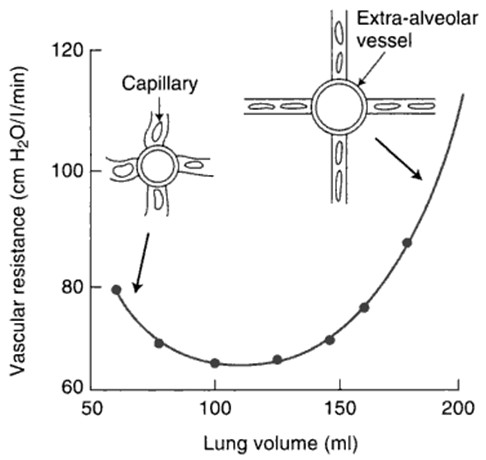
**Increased PVR at extremes of lung volumes**. This figure represents measurements made in an animal-lobe preparation in which the transmural pressure of the capillaries is held constant. It illustrates that at low lung volumes (as may occur with atelectasis), extraalveolar vessels become narrow, and smooth muscle and elastic fibers in these collapsed vessels increase PVR. At high lung volumes, as alveolar volumes are increased and walls are thinned, capillaries are stretched, reducing their caliber and also increasing PVR. (Adapted from John West's *Essential Physiology*, 10^th ^edition, Philadelphia: Lippincott & Williams, with permission).

PDE5 inhibitors, including sildenafil and vardenafil, increase downstream cGMP signaling, potentiating the beneficial effects of NO (Figure 4). PDE5 inhibitors acutely reduce PVR [308,309], and increase CO and reduce PAOP more than does NO [310]. These agents improve clinical end-points in PAH [311], where endothelial NO is reduced [312] and PDE5 expression is upregulated [313,314]. PDE5 inhibitor may also exert milrinone-like effects through PDEIII inhibition, augmenting RV function [310,311,315]. Despite their relative pulmonary selectivity and rapid onset, however, adverse effects may include reduced SVR with potential effects on RV performance [316]. Oral sildenafil has been used to reduce PVR effectively in well-selected patients with PH after cardiac surgery without reducing the SVR [269,317-319]. Even a single dose may facilitate weaning from NO [266], also without reducing SVR [266-269]. Sildenafil may also improve myocardial perfusion and reduce platelet activation [320] as well as endothelial dysfunction after CPB [321]. Oral sildenafil has been effective in patients with PH due to left ventricular systolic dysfunction, reducing PVR and increasing CO, although reducing the SVR [260]. Sildenafil has also been used in selected patients with PH due to selected cases of chronic respiratory disease without worsening oxygenation or SVR [322,323]. A single dose of 50 mg nasogastric sildenafil has been studied in a small cohort of consecutive ARDS patients, lowering MAP, and worsening oxygenation due to increased V/Q mismatch, although RV performance did improve [324]. Intravenous sildenafil has been shown to reduce SVR and PVR in end-stage congestive heart failure patients [325], although it is not available commercially, and its use is not licensed in unstable patients (Table 6).

### GRADE RECOMMENDATION 7

Based on mostly moderate-quality evidence (see Additional file 1), the following STRONG recommendation is made: pulmonary vasodilators reduce PVR, improve CO and oxygenation, and may be useful when PH and RV dysfunction are present, notably after cardiac surgery.

Based on mostly moderate-quality evidence (see Additional file 1), the ICU side-effect profile of intravenous pulmonary vasodilators may be less favorable than that of inhaled agents. The following STRONG recommendation is therefore made: Consideration should be given to the use of inhaled rather than systemic agents when systemic hypotension is likely, and concomitant vasopressor use should be anticipated.

Based on mostly high-quality evidence (see Additional file 1), the following STRONG recommendation is made: give consideration for the use of NO as a short-term therapy to improve oxygenation indices but not outcome in patients with ARDS. Based on low-quality evidence (see Additional file 1), a WEAK recommendation is made that pulmonary vasodilators may also be useful treat PH associated with RV dysfunction in ARDS.

Based on mostly low-quality evidence (see Additional file 1), the following WEAK recommendation is made: Oral sildenafil may reduce PVR and facilitate weaning from NO after cardiac surgery in selected patients with PH, without adverse effects on systemic blood pressure in well-selected patients.

### Nonpharmacologic Management

This encompasses RV "protective" strategies to avoid factors (Table 3) that may further increase PVR. Mechanical devices are also increasingly used to give a failing RV a bridge to recovery or transplantation.

#### Ventilatory strategies

Important variables that may reduce pulmonary blood flow during ventilation include hypoxia, hypercapnia, and compression of the pulmonary vasculature at the extremes of lung volumes (Figure 4). Acute hypoxia leading to hypoxic pulmonary vasoconstriction is well described [326] and may be augmented by many factors, including acidosis [327]. Acute hypercapnia also leads to pulmonary vasoconstriction [328,329], although this may be attenuated with NO [330], and, when associated with high PEEP, leads to RV dilatation and reduced cardiac output in severe ARDS [328,329]. A reduction in pulmonary blood flow occurs both at low volumes, such as in areas of atelectasis, and at high lung volumes, such as with increased airway plateau pressure (P_plat_): Increased RV afterload, reduced venous return, and acute RV dysfunction may result [331]. Both atelectasis and ventilation at high lung volumes should therefore be avoided in patients with RV dysfunction.

Before the era of protective ventilatory strategies in ARDS, the incidence of acute RV failure was 60% [332] and has since decreased to 10% to 25% [24]. This is thought to reflect the change in ventilatory practice: lower P_plat _reduces the incidence of RV failure [333]. Prone ventilation may also reduce P_plat _and pCO_2 _sufficiently to improve acute RV failure [334]. In ARDS, transition to high-frequency oscillation leads to an increase in CVP and a minor decrease in cardiac output due to preload reduction [335], and RV function may decrease during recruitment maneuvers [336]. In children after Fontan procedures, the hemodynamic effects of negative-pressure ventilation (NPV) are nicely illustrated by measuring pulmonary blood flow: after a switch from conventional intermittent positive pressure ventilation (IPPV) to NPV by using cuirass ventilation, pulmonary blood flow, stroke volume, and cardiac output increased up to 50%, and decreased to baseline when IPPV was reinstituted [337,338].

#### Mechanical support

Mechanical support for the RV may be appropriate in reversible settings or as a bridge to definitive treatment. RV-assist devices (RVADs) may be used in primary RV dysfunction [339] and have been used with coexisting PH [340,341]. There is, however, concern that pulsatile devices may cause pulmonary microcirculatory damage in PH [342,343]. A pumpless "lung assist" device has been used in patients bridging to transplant [344]. Extracorporeal membrane oxygenation (ECMO) has been used in severe PH [345-348], as a bridge to transplant [349,350], and after endarterectomy [351] or massive PE [352-355]. Intraaortic balloon counterpulsation (IABP) has been used for RV failure after CPB [356] and transplantation [357], thought to improve CO by augmenting left coronary flow rather than by direct RV effects [358]. Atrial septostomy creates a right-to-left shunt that improves left atrial filling and LV function while reducing RV end-diastolic pressure and improving RV contractility. It is sometimes used as a bridge to transplantation in severe PAH [359], although not in patients with very severe RV failure [360].

### GRADE RECOMMENDATION 8

Based on mostly very-low-quality evidence, the following WEAK recommendation is made: Mechanical therapies including ECMO and IABP may have a role as rescue therapies in reversible pulmonary vascular dysfunction or while awaiting definitive treatment.

## Conclusions

Pulmonary vascular and right ventricular dysfunction may complicate many ICU illnesses: the diagnosis may be difficult, and the acute management, challenging. Their presence is associated with a worse outcome. This review highlights that some recommendations can be made, despite limitations of the GRADE analysis. However, we do consider that "weak GRADE recommendations" could be interpreted as "management suggestions" and treated with appropriate caution. A further limitation is that several pathologies have been grouped together as one syndrome, although this relates to both the rarity of the syndrome and the lack of high-quality evidence: further research is desperately needed. In particular, only then will we learn whether PAH-targeted therapy such as use of PDE5 inhibitors or endothelin-receptor antagonists, so effective in idiopathic PAH, have a role in the ICU setting.

## Key messages

• Pulmonary hypertension (PH) and associated right ventricular (RV) failure are associated with worse outcomes in critical care, and because of nonspecific presenting symptoms and signs, may be difficult to recognize: echocardiography is a very useful initial test, and invasive monitoring may be helpful in some cases for more continuous monitoring and accurate measurement of pulmonary vascular resistance.

• Volume loading of the right ventricle may worsen its performance: all fluid challenges should be closely monitored.

• It is essential to maintain adequate aortic root pressure to prevent the onset of RV ischemia. Vasopressors are useful in this setting, including low-dose norepinephrine as a first-line agent. Low-dose vasopressin may also be useful in some resistant cases but has adverse myocardial effects at higher doses. Potentially useful inotropes in RV failure include dobutamine and those with additional pulmonary vasodilating effects, including PDE III inhibitors, although co-administration with pressors is often necessary. The effects of any vasoactive drug may be unpredictable in an individual and require close clinical observation of circulatory performance, potentially assisted by echocardiography.

• Pulmonary vasodilators are useful to reduce RV afterload in several ICU settings, including PH and RV failure after cardiac surgery. Systemic administration may worsen systemic hemodynamics and oxygenation because of ventilation-perfusion mismatching.

• The use of mechanical therapies to manage acute PH and enhance RV performance is expanding, although with evidence currently limited to case series, and may be useful in experienced centers to ameliorate RV failure while awaiting definitive therapy.

## Abbreviations

ACP: acute cor pulmonale; ARDS: acute respiratory distress syndrome; AVP: arginine vasopressin; cAMP: cyclic adenosine 3',5'-cyclic monophosphate; cGMP: cyclic guanosine 3',5'-cyclic monophosphate; CI: cardiac index; CO: cardiac output; COPD: chronic obstructive pulmonary disease; CPB: cardiopulmonary bypass; CVP: central venous pressure; ECMO: extracorporeal membrane oxygenation; ICU: intensive care unit; IABP: intraaortic balloon pump; LV: left ventricle; MVR: mitral valve replacement; NE: norepinephrine; NO: nitric oxide; PAC: pulmonary artery catheter; PAH: pulmonary arterial hypertension; PAOP: pulmonary arterial occlusion pressure; PDE: phosphodiesterase; PE: pulmonary embolism; PGE_1_: prostaglandin E_1_; PH: pulmonary hypertension; PHE: phenylephrine; PVR: pulmonary vascular resistance; RV: right ventricle; RVEF: right ventricular ejection fraction; RVF: right ventricular failure; SvO_2_: mixed venous oxygen saturation; SVR: systemic vascular resistance; TEE: transesophageal echocardiography; TR: tricuspid regurgitation; V/Q mismatch: ventilation/perfusion mismatch.

## Competing interests

LCP has received honoraria from Encysive Pharmaceuticals. SJW has received honoraria from Actelion Pharmaceuticals. SJB has received support for clinical trials from Pfizer, Astra Zeneca, and Baxter Healthcare.

## Authors' contributions

LCP and SJB conceived of the review and participated in its design. LCP and SJW carried out the literature search and drafted the initial manuscript. All authors read and approved the final manuscript.

## Supplementary Material

Additional file 1 **Population, Intervention, Comparison and Outcome (PICO) evidence tables**. This file contains structured detail for all studies included in the systematic review. According to GRADE method guidelines, a series of eight study questions was devised to approach the questions posed by the systematic literature review. The PICO table then describes each study according to the study type, the population studied, the intervention applied, the nature of the comparison or control group, and the studied outcome of interest appropriate to the study question. The final column grades the evidence according to the GRADE evidence level as very low-, low-, moderate-, or high-level evidence [80,81]. Click here for file
