# Supplementary material for: Pulmonary vascular and right ventricular dysfunction in adult critical care: current and emerging options for management: a systematic literature review
Source: Crit Care. 2010 Sep 21;14(5):R169. doi: 10.1186/cc9264 (PMC3219266; doi:10.1186/cc9264)
Supplement: Additional file 1 — Population, Intervention, Comparison and Outcome (PICO) evidence tables. This file contains structured detail for all studies included in the systematic review. According to GRADE method guidelines, a series of eight study questions was devised to approach the questions posed by the systematic literature review. The PICO table then describes each study according to the study type, the population studied, the intervention applied, the nature of the comparison or control group, and the studied outcome of interest appropriate to the study question. The final column grades the evidence according to the GRADE evidence level as very low-, low-, moderate-, or high-level evidence [80,81]. [file cc9264-S1.DOC]

**Additional File 1: Population, Intervention, Comparison & Outcomes (PICO) Evidence Tables**

**Structured Clinical Question 1:** Management of volume status in patients with pulmonary vascular dysfunction

**PICO 1: Volume status in pulmonary vascular dysfunction**

| **Patient Population** | | **Intervention** | | | **Comparison** | | **Outcomes** | | | **Comments** | | |
| --- | --- | --- | --- | --- | --- | --- | --- | --- | --- | --- | --- | --- |
| Adult ICU patients at risk of developing RV dysfunction / failure as a result of a period of elevated PVR.  Including:   - Adults receiving post-operative care for cardiothoracic and transplantation surgery - Patients with known pre-existing PH undergoing surgery (including obstetric procedures) - Other clinical subgroups at risk of PVD including acute PE, ARDS/ALI, sepsis. | | Management of fluids and volume status in adult patients with RV functional impairment due to elevated PVR during critical illness.  Search terms:   - PH - ICU - RV failure - Fluid therapy - Volume status | | | Reference standard (well validated assessment tools/gold standard that are used for confirming diagnosis) | | - Improved cardiovascular status - Improved RV function (outcome measures including SV, RVEF,CO, MAP) - Morbidity (adverse effects) - Any other outcomes e.g. ICU LOS - Mortality | | | Study design:  Cross-sectional studies, case-control studies, cohort studies, RCTs.  Date limit: since 1980.  Limited to studies reported in English. | | |
| **Summary of evidence***. NB* We were unable to find any other studies on diuresis or haemofiltration in setting of RV failure due to PH. | | | | | | | | | | |  | |
| **Study author (ref)** | **Study type** | | **Population** | **Intervention** | | **Comparison** | | **Outcome of interest** | **Comments (GRADE level)** | | |  |
| *Redl[93]*. | Cohort study | | 27 septic shock patients, 41% with RV dysfunction. PAC. | Infusion therapy until max achievable MAP/CI. | | None | | All patients needed catecholamines in addition to fluids to achieve haemodynamic stability. | LOW | | |  |
| *Schneider* [94]. | Cohort study | | 18 septic shock patients with PH. RVEF, LVEF. PAC. | Infusion therapy. | | None | | Patients who did not increment SVI had higher baseline CVP and lower MAP associated with RV failure and coronary hypotension. | LOW | | |  |
| *Mercat [92]* | Cohort study | | 13 patients with acute cor pulmonale following PE. | Infusion of 500ml dextran | | None | | RAP increased (9 to 17mmHg, p<0.05), CI increased (1.6 t o 2.0, p<0.05), RVEF and PVR unchanged | LOW | | |  |
| *Ferrario[151]*. | Non-randomised controlled study | | 11 patients with RV infarction, high CVP & low CO. | Volume loading (400ml NS) | | Dobutamine (up to 10mcg/kg/min) | | Volume increased RAP, PAWP, with no improvement in CI; Dobutamine increased CI (both HR and SVI) and myocardial performance. | LOW | | |  |
| *Siva[95]*. | Case report | | 27yr-old pregnant woman with mitral stenosis, severe PH and RV pressure overload. | Diuretics | | None | | Oral diuretics improved RV function. | VERY LOW | | |  |

**Abbreviations**: RCT randomised controlled study, PAC pulmonary artery catheter, MF main finding, RVF right ventricular ejection fraction, LVEF left ventricular ejection fraction, CVP central venous pressure, NS normal saline, RAP right atrial pressure, PAWP pulmonary artery wedge pressure, CI cardiac index, HR heart rate, SV(I) stroke volume (index), PAP pulmonary artery pressure.

**Structured Clinical Questions 2-3:** The effectiveness of vasopressors to augment SVR, with minimal adverse effects, in patients with pulmonary vascular dysfunction

**PICO 2&3: Vasopressors in pulmonary vascular dysfunction**

| **Patient Population** | | **Intervention** | | | **Comparison** | | **Outcomes** | | | **Comments** | |
| --- | --- | --- | --- | --- | --- | --- | --- | --- | --- | --- | --- |
| Adult ICU patients at risk of developing RV dysfunction / failure as a result of a period of elevated PVR.  Including:   - Adults receiving post-operative care for cardiothoracic and transplantation surgery - Patients with known pre-existing PH undergoing surgery (including obstetric procedures) - Other clinical subgroups at risk of PVD including acute PE, ARDS/ALI, sepsis. | | The clinical effectiveness of vasoactive agents in PVD  Search terms:  *Vasopressors:*   - phenylephrine (PHE) - norepinephrine (NE) - Arginine vasopressin (AVP) | | | ‘Standard usual care’ | | - Effects on RV function (measures including SV, RVEF,CO, MAP) - Effects on PVR or PVR/SVR - Effects on heart rate - Morbidity: any adverse events - Effects on CPB weaning - ICU length of stay - ICU Mortality | | | Study design:  RCTs, cohort studies, case control studies, case series, case reports.  Date limit: since 1980.  Limited to studies reported in English. | |
| **Summary of evidence. Vasopressors: norepinephrine (NE), phenylephrine (PHE)**; **vasopressin (AVP)**: | | | | | | | | | | |  |
| **Study author (ref)** | **Study type** | | **Population** | **Intervention** | | **Comparison** | | **Outcome of interest** | **Comments (GRADE level)** | |  |
| *Morelli[105]* | RCT pilot | | n=45 hypotensive septic shock patients. PAC. | NE (15mcg/min) | | TLP, AVP | | NE increased PVR & PVR/SVR compared to AVP and TLP. | MODERATE. | |  |
| *Martin[109]*. | Prospective DB RCT. PAC. | | 9 hypotensive septic shock with PH. | Volume then NE (0.5-5mcg/kg/min) to restore MAP. | | Volume then dopamine (2.5-25mcg/kg/min) to restore MAP. | | NE increased MAP and PVRI. Despite increased RV afterload, RVEF and RV performance. | MODERATE | |  |
| *Schreuder[106]*. | Crossover study | | n=10 septic shock patients with PH and RV dysfunction needing vasoactive drugs after fluid replacement. | Dopamine | | NE | | NE increased SVR and PVR but improved RV oxygen supply/demand ratio. | LOW | |  |
| *Tritapepe[365]*. | Self-controlled study | | 10 non-consecutive patients post-CPB with refractory PH and low CO. | Given biatrial low-dose RA PGE1 (31.5ng/kg/min)  and LA NE (0.11mcg/kg/min). | | Baseline | | Combination caused dramatic reduction in PVR, PVR/SVR and increased CI. | LOW. | |  |
| *Bertolissi[110]*. | Case report. | | 48y old woman emergency mitral valve surgery, post-op PH and RV failure. | NE | | None | | NE increased CI without adverse effect on PAP. | VERY LOW. | |  |
| *Tourneux[104]*. | Self-controlled study | | 18 infants with PPHN and circulatory failure treated with NO after fluid resuscitation. Echo. | NE (0.5mcg/kg/min) | | Baseline | | NE increased MAP and LV CO; NE also reduced PVR/SVR. | MODERATE | |  |
| *Kwak[100]*. | Randomise clinical trial. | | 27 Patients with chronic PH mostly due to mitral valve disease with hypotension following anaesthesia | NE infused to increment MAP by 30-50%. | | PHE infused to increment MAP by 30-50%. | | NE reduced PVR/SVR without a change in CI. PHE less effective as a pressor; reduced CI and did not reduce PVR/SVR. | NE preferable to PHE in patients with chronic PH. LOW | |  |
| *Rich[111]*. | Self-controlled study | | 10 patients with PH. | PHE infusion | | Baseline | | Increased coronary driving pressure but worsened RV function (increased in RVEDP; fall in CO). | LOW. | |  |
| *Jeon[115]*. | RCT. | | n=50 post-off-pump CABG patients with milrinone-induced hypotension. PAC. | Randomised to NE or AVP (0.02-0.16u/min). | | AVP | | Both agents increased MAP. Only AVP-milrinone reduced PVR/SVR (no effect on PVR/SVR with NE-milrinone). | Both NE and MIL effectively reverse milrinone-induced hypotension post-CABG, but only AVP-MIL reduced PVR/SVR. MODERATE. | |  |
| *Tayama[116]*. | Self-controlled study | | 9 cases of post-cardiotomy catecholamine-resistant hypotension with PH, despite NO and IABP. | AVP (0.05-0.1u/min) | | Baseline | | AVP increased SVR; PVR unchanged but PVR/SVR lowered. Slight drop in CI (NS). UO increased. | LOW | |  |
| *Morelli[105]*. | RCT pilot. | | n=45 septic shock patients. PAC. | NE | | TLP, AVP (0.03u/min) | | AVP (& TLP) reduced PVR & PVR/SVR. | MODERATE. | |  |
| *Braun[117]*. | Case report | | Patient with PAH following GA-induced hypotension. | AVP | | None | | Successful increment in MAP without adverse effects. | VERY LOW. | |  |
| *Price[118]*. | Case report | | PH crisis following Caesarean section in 2 patients with IPAH. PAC/CVP. | AVP | | None | | Increment in MAP achieved and reversal of PH crisis without adverse PVR/RV effects. | VERY LOW. | |  |
| *Argenziano[124]*. | RCT | | Post-CPB shock. n=23. | AVP (0.1u/min) | | Saline placebo. | | AVP increased SVR, decreased NE requirements | MODERATE | |  |
| *Torgersen[121]*. | Non-randomised controlled trial | | 50 patients with vasodilatory shock due to sepsis, SIRS or post-cardiac surgery requiring NE>0.6mcg/kg/min | AVP 0.033 u/min | | AVP 0.067u/min. | | MAP restored, heart rate reduced in both groups.NE requirements even less in 0.067u/min group. Equivalent adverse events. | AVP 0.067u/min restores haemodynamic function in advanced vasodilatory shock better than 0.033u/min. MODERATE. | |  |
| *Scheurer[126]*. | Case report | | 2 cases of PH following corrective cardiac surgery. | AVP | | None | | AVP used to improve MAP and reduce PAP | VERY LOW. | |  |
| *Vida[125]*. | Case report | | 33y-old woman post-cardiac surgery (to repair congenital malformations). | AVP | | None | | AVP used for catecholamine-resistant hypotension: improved haemodynamics; allowed weaning of inotropes. | VERY LOW. | |  |
| *Wang[127]*. | Case report | | Septic shock in patient with chronic PH and catecholamine-resistant hypotension | AVP | | None | | AVP reduced PH and led to haemodynamic stabilisation. | VERY LOW. | |  |
| *Migotto[136]*. | Case series | | 8 patients with cardiogenic shock | AVP (0.02-0.5u/min). | | None | | CI increased in all except 3/8 when doses>0.08u/min used reduced CI. | VERY LOW. | |  |
| *Jain[128]*. | Case series | | 19 patents with septic shock and pre-existing PAH. 7 had PAC. | AVP (0.04u/min) | | Baseline | | AVP was effective and safe in most patients. Stable mPAP & SvO2 after AVP. 8 deaths in group due to progressive RVF not felt to be attributable to AVP. | VERY LOW. | |  |
| *Holmes[129]*. | Case series | | 50 patients with septic shock receiving AVP: | AVP | | Baseline | | Adverse cardiac effects seen at doses >0.04u/min. | LOW. | |  |
| *Dunser[366]*. | Self-controlled study | | 60 patients with septic / post-cardiotomy shock. | AVP | | Baseline | | AVP increased MAP, SVR, reduced HR and mPAP with unchanged SVI. | LOW. | |  |
| *Dunser[131]*. | Self-controlled study | | 41 post-cardiotomy shock patients. PAC. | AVP (0.067u/min). | | Baseline | | AVP decreased HR, milrinone & NE requirements, increased LVSWI, MAP AND SVR. 45% new-onset tachyarrhythmias cardioverted to sinus rhythm with AVP. Reduction in cardiac enzymes with AVP. | LOW. | |  |
| *Dunser[130]*. | Prospective RCT | | 48 patients with catecholamine-resistant vasodilatory shock (post-cardiac surgery, SIRS, and sepsis). PAC. | AVP (0.067u/min) combined with NE | | NE alone | | NE-alone group had higher heart rates and got more new-onset tachyarrhythmias. AVP group had preserved GI perfusion and better myocardial performance than NE group. | MODERATE. | |  |
| *Lauzier[132]*. | RCT | | 23 patients with early (12h) hyperdynamic septic shock. | AVP (0.04-0.2u/min) | | NE single agent (0.1-2.8mcg/kg/min) for 48h to achieve MAP>70mmHg | | AVP alone similarly increased MAP at 48h, but less than NE in 1^st^ hour; high AVP doses reduced CI (by reducing HR) and led to ACS in 1 patient with dose-dependent ECG changes. | MODERATE. | |  |
| *Naeije[134]*. | Non-randomised controlled study | | 18 patients with cirrhosis. | AVP (0.8u/min) given to 8 patients. | | Somatostatin | | AVP led to persistent rise in PVR + SVR (although PVR/SVR fell); bradycardia and negative effects on CO and oxygen delivery. | LOW. | |  |
| *Mols[135]*. | Crossover study | | 12 cirrhotics. PAC. | AVP (0.4u/min) alone | | AVP with SNP (1-5mcg/kg/min) | | AVP reduced CO and O2 delivery by 25%, increased MAP (by 20%) and PAWP, reduced portal pressures. | LOW. | |  |

**Abbreviations** (in addition to list in table 1): TLP terlipressin, AVP arginine vasopressin, CPB cardiopulmonary bypass, SIRS systemic inflammatory response syndrome, SNP sodium nitroprusside, PH pulmonary hypertension, PPHN persistent PH of newborn, PAH pulmonary arterial hypertension, CABG cardiopulmonary bypass

**Structured Clinical Questions 4-6:** The effectiveness of inotropes to augment CI, with minimal adverse effects, in patients with pulmonary vascular dysfunction

**PICO 4, 5, 6: Inotropes in pulmonary vascular dysfunction**

| **Patient Population** | | **Intervention** | | | **Comparison** | | **Outcomes** | | | **Comments** | |
| --- | --- | --- | --- | --- | --- | --- | --- | --- | --- | --- | --- |
| Adult ICU patients at risk of developing RV dysfunction / failure as a result of a period of elevated PVR.  Including:   - Adults receiving post-operative care for cardiothoracic and transplantation surgery - Patients with known pre-existing PH undergoing surgery (including obstetric procedures) - Other clinical subgroups at risk of PVD including acute PE, ARDS/ALI, sepsis. | | The clinical effectiveness of inotropes in PVD  Search terms:  *Sympathomimetic Inotropes:*   - Dobutamine - Dopamine - Epinephrine   *Inodilators*:   - PDE3 inhibitors   Milrinone, amrinone, enoximone   - Levosimendan | | | ‘Standard usual care’ | | - Effects on RV function (measures including SV, RVEF,CO, MAP) - Effects on PVR or PVR/SVR - Effects on heart rate - Morbidity: any adverse events - Effects on CPB weaning - ICU length of stay - ICU Mortality | | | Study design:  RCTs, cohort studies, case control studies, case series, case reports.  Date limit: since 1980.  Limited to studies reported in English. | |
| **Summary of evidence: Sympathomimetic** **inotropes; inodilators.** | | | | | | | | | | |  |
| **Study author (ref)** | **Study type** | | **Population** | **Intervention** | | **Comparison** | | **Outcome of interest** | **Comments (GRADE level)** | |  |
| *Holloway[141]*. | Self-controlled study | | 10 PH patients | Pre-dopamine | | Post-dopamine | | HR and CO increased, mPAP increased, PVR unchanged. | LOW. | |  |
| *Liet[142]*. | Self-controlled study | | n=18 hypotensive neonates with patent ductus arteriosus | Pre-dopamine | | Post-dopamine | | Dopamine variable effects: 50% increased PVR/SVR following dopamine. | LOW (neonates) | |  |
| *Leier[143]*. | Crossover study | | 13 patients with cardiomyopathy using PAC. | Dopamine | | Dobutamine (2.5-10mcg/kg/min) | | Dobutamine increased SV and CO while reducing SVR & PVR, without a change in HR. Dopamine increased HR at >6mcg/kg/min. | MODERATE. | |  |
| *Le Tulzo[14]*. | Self-controlled study | | 14 consecutive patients with septic shock and RVF using PAC. | Before epinephrine | | After epinephrine | | EPI increased MAP, CI, SVI without change in PAWP or HR. EPI did raise mPAP but improved RVEF. | LOW. | |  |
| *Acosta[150]*. | Non-randomised controlled trial | | Cirrhotic patients at OLT with and without PH. Comparison of dobutamine on RV function and RV-arterial coupling.  PAC. | Patients with cirrhosis and PH given dobutamine 5-10mcg/kg/min) prior to anhepatic phase, to maintain CI>3l/min/m2 | | Patients with cirrhosis and no PH given dobutamine 5-10mcg/kg/min) | | RV contractility and afterload higher in PH group. In both groups, dobutamine increased RV contractility and reduced afterload (i.e. V-A coupling increased). | LOW. | |  |
| *Ferrario[151]*. | Non-randomised crossover trial | | 11 patients with RV infarction with high CVP & low CO. PAC. | Dobutamine (up to 10mcg/kg/min) | | Volume loading (400ml NS) | | Volume increased RAP, PAWP, with no increase in CI; Dobutamine inc’d CI (both HR and SVI) and myocardial performance. | MODERATE. | |  |
| *Vizza[153]*. | Crossover study | | 28 lung transplant candidates. PAC. | Dobutamine | | NO. both | | Dobutamine increase CI, and reduced pO2 (due to increase in shunt fraction). When combined with NO, further increase in CI and improved pO2. | MODERATE. | |  |
| *Eichorn[164]*. | Randomised controlled study | | 14 patients with CCF | Intravenous milrinone | | Dobutamine | | Both incremented CI similarly without change in HR. Both increased RVEF. PVR reduced by milrinone more than dobutamine. | MODERATE. | |  |
| Harris[163]. | Multicentre RCT | | 99 adults undergoing cardiac surgery with post-op CI<2.5l/min/m2. Some patients (for MVR surgery) had high baseline PVR | Milrinone bolus then infusion at incremental doses | | Comparing to baseline and different doses | | Milrinone progressively reduced PVR (initially mPAP fell by 15% then max effect seen at 12h), especially early on in MVR group with highest baseline PVR, with increase in CI in all patients. | HIGH. | |  |
| *Kihara[161]*. | Case reports | | 2 cases of RVF following LVAD insertion. | Milrinone | | None | | MIL reduced PVR and improved LVAD flow. | VERY LOW. | |  |
| *Kikura[173]*. | Randomised controlled study | | 27 patients at CPB | Milrinone | | Control (no milrinone) | | To assess effect on platelet function using bleeding time (BT), coagulation samples and TEG. Milrinone did not change parameters compared to usual post-CPB effects (reduced platelet count, increased BT, no TEG changes). | MODERATE. | |  |
| *Oztekin[160]*. | Randomised controlled trial | | 47 patients with PH (due to left-heart disease) having MVR for rheumatic MS. PAC. | Milrinone (MIL) loading dose then infusion during weaning from CPB until end of surgery | | Control (no milrinone) | | PAP, CVP, PAWP lower in MIL group; Control group required higher doses vasodilators, inotropes, anti arrhythmias, diuretics & fluid restriction. Post-op MAP lower in MIL group, although no increase in vasopressor use. | MODERATE. | |  |
| Fukazawa[162]. | Case report | | Portopulmonary hypertension at OLT, us, PAC/TOE. | Milrinone | | None | | effective at controlling high perioperative PAP. | Very low | |  |
| *Tarr[166]*. | Case series | | (n=10) of post-MVR low CO with PH. | Enoximone | | None | | Significant reduction SVR, PVR, sustained increase in CI. Successfully weaned from CPB. | Very low | |  |
| *Boldt[165]*. | Randomised study | | 40 post-CPB with low CO | Enoximone pre-weaning | | No enoximone pre-weaning | | Doppler skin blood flow (no PAC) showed an increase in skin blood flow; fewer enoximone patients needed epinephrine; more needed NE. | LOW. | |  |
| *Leeman[167]*. | Self**-** controlled study | | 19 patients with decompensated COPD. PAC. | Pre-enoximone | | Post**-** enoximone | | Reduced PCWP, RAP, mPAP, MAP. CO unchanged. HR increased slightly. | LOW. | |  |
| *Dupuis[169]*. | Randomised controlled study | | 30 patients with low CO post CPB. | Amrinone | | Dobutamine (5-15mcg/kg/min) | | No difference in haemodynamics between groups treated with each agent alone. 6 (40%) dobutamine had post-op MI compared to none with amrinone. | MODERATE. | |  |
| *Jenkins[168]*. | DB RCT | | 20 patients undergoing MVR | Dobutamine (5mcg/kg/min) | | Amrinone (10mcg/kg/min). | | Amrinone increased CI and RVEF & reduced PAWP and PAP more than dobutamine. Similar effects on MAP, HR, CVP and RVSWI. | MODERATE | |  |
| *Hachenberg[170]*. | Randomised controlled study | | 20 patients having MVR (for mitral regurgitation). | Enoximone | | Dobutamine | | Enoximone increased CI, HR, decreased PAP post-CPB more than dobutamine, and enoximone caused less deterioration of gas exchange. | MODERATE | |  |
| *Ansell[171]*. | Cohort study | | Thrombocytopenia assessed in 43 patients receiving amrinone | Amrinone | | No amrinone | | Thrombocytopenia mostly mild, without attributable bleeding in most cases. | LOW. | |  |
| *Jeon[115]*. | RCT | | 50 post off-pump CABG patients with milrinone-induced hypotension. PAC. | NE | | AVP (0.02-0.16u/min) | | Both agents increased MAP. Only AVP-milrinone reduced PVR/SVR (no effect on PVR/SVR with NE-milrinone). | MODERATE. | |  |
| *Haraldsson[178]*. | Non-randomised controlled study | | 20 patients with post-operative PH and RV dysfunction post cardiac surgery. | Inhaled milrinone | | Inhaled milrinone-PGI2 | | Inhaled milrinone reduced PVR; PGI2+MIL further and prolonged PVR reduction and increased SV. SVR not affected by either combination. | MODERATE. | |  |
| *Sablotzki[179]*. | Self-controlled study | | 18 consecutive heart transplant candidates (9 with PH). | Inhaled milrinone (2mg nebulised) | | Baseline | | Reduced PVR in patients with PH. no effect on SVR. | MODERATE. | |  |
| *Buckley[176]*. | Case report | | 42-yr-old woman with PH crisis due to decompensated IPAH, already on iv treprostinil, NO, dobutamine, AVP, epinephrine. | Inhaled milrinone | | None | | Inhaled MIL added as salvage therapy for 8d. PAH symptoms improved, with no compromise on SVR or HR. | VERY LOW. | |  |
| *Wang[177]*. | Randomised study | | 48 patients with PH post-MVR surgery. PAC. | Intravenous milrinone | | Inhaled milrinone | | PVR decrease similar. SVR maintained better in inhaled group. Reduced shunt fraction in inhaled group. | MODERATE. | |  |
| *Slawsky[185]* | DBRCT | | 146 NYHA III-IV patients with LV dysfunction. | Levosimendan | | Placebo | | Levosimendan increased SV, CO, HR. Levosimendan reduced PCWP, RAP, PAP, MAP. | HIGH (in patients with left-sided heart disease) | |  |
| *Parissis[194]* | RCT | | 54 patients with LV systolic dysfunction. Assessment of effects on RV using echo tissue Doppler. | Levosimendan | | Placebo | | Levosimendan increased maximal systolic tricuspid velocity (S wave) and reduced PAP compared to placebo. | MODERATE. | |  |
| *Duygu[190]* | RCT | | Patients with acute heart ischaemic failure and LVEF<40%. Assessment of effects on RV using echo tissue Doppler. | Levosimendan | | Dobutamine | | Levosimendan increased tricuspid velocities and reduced SPAP, whereas dobutamine although reduced sPAP (to a lesser degree), did not augment RV function. | Levosimendan improves RV systolic and diastolic function. MODERATE | |  |
| *Poezl[193]* | Self-controlled study. | | 18 patients with acute heart failure (PCWP>15mmHg) predominantly due to RV failure. PAC. | Levosimendan for 24h | | Baseline | | LWSWI increased, PCWP, SVR and RAP decreased with levosimendan. Decrease in PAP and PVR not significant. RVSWI increased. | Haemodynamic improvement in RVF. Possibly through increased RV contractility than reduced afterload. LOW. | |  |
| *Russ[191]* | Observational non-randomised study | | 56 consecutive patients with cardiogenic shock following MI, after revascularization, inotropes +/- IABP. Looking at RV performance. | Levosimendan | | Conventional therapy (dobutamine, norepinephrine) | | Levosimendan improved RV performance; increased in CI, increased RV power index, and reduction in PVR. | LOW | |  |
| *Yilmaz[192]* | RCT | | 40 consecutive patients with severe decompensated biventricular failure. Echo. | Levosimendan | | Dobutamine | | Both improved EF and reduced SPAP. Levosimendan improved systolic RV function more than dobutamine. | LOW. | |  |
| *Morelli[195]* | RCT | | 35 ARDS and septic shock patients. MAP 70-80mmHg sustained with NE. | Levosimendan | | Placebo | | Levosimendan increased CI, decreased PVR and PAP, increased RVEF, and SvO2. | Levosimendan improves RV performance through pulmonary vasodilator effects in septic ARDS patients. MODERATE. | |  |
| *Morais[196]* | Case report | | 33-yr-old woman with RVF following emergency MVR surgery for severe decompensated mitral stenosis | Difficult CPB weaning with suprasystemic PAP. Levosimendan added to milrinone, epinephrine. | | None | | Addition of levosimendan stabilised haemodynamics post-CPB | VERY LOW. | |  |
| *Cicekcoglu[197]* | Case report | | 2 patients with PH undergoing mitral valve surgery. | Levosimendan | | None | | Levosimendan tolerated perioperatively and reduced PH. | VERY LOW. | |  |
| *Kleber[198]* | RCT | | 28 patients with chronic PH (mostly PAH and PH due to left-heart disease; Mostly NYHA III-IV) | Levosimendan | | Placebo | | Initial significant decrease in PVR and PAP. SvO2 increased. maintained at 8 weeks (i.e. no tolerance) | LOW. | |  |

**Abbreviations**: OLT orthotopic liver transplantation, MIL milrinone, TOE transoesophageal echo, PVR pulmonary vascular resistance, SVR systemic vascular resistance, LVAD left ventricular assist device, MVR mitral valve replacement, DB double-blind, TEG thromboelastography, NO nitric oxide.

**Structured Clinical Question 7:** The effectiveness of pulmonary vasodilators to reduce PVR, and improve RV function, with minimal adverse effects, in patients with pulmonary vascular dysfunction

**PICO 7: Pulmonary vasodilators in pulmonary vascular dysfunction**

| **Patient Population** | | **Intervention** | | | **Comparison** | | **Outcomes** | | | **Comments** | |
| --- | --- | --- | --- | --- | --- | --- | --- | --- | --- | --- | --- |
| Adult ICU patients at risk of developing RV dysfunction / failure as a result of a period of elevated PVR.  Including:   - Adults receiving post-operative care for cardiothoracic and transplantation surgery - Patients with known pre-existing PH undergoing surgery (including obstetric procedures) - Other clinical subgroups at risk of PVD including acute PE, ARDS/ALI, sepsis. | | The clinical effectiveness of pulmonary vasodilators in PVD in ICU setting  Search terms:  Prostanoids*:*   - prostacyclin (PGI2, Flolan, epoprostenol): intravenous; inhaled - PGE1 - iloprost   Inhaled NO  PDE5 inhibitors   - Sildenafil; vardenafil   ERAs   - Bosentan | | | ‘Standard usual care’ | | - Effects on RV function (measures including SV, RVEF,CO, MAP) - Effects on PVR - Morbidity: any adverse events - Effects on CPB weaning - Effects on weaning ventilation - ICU length of stay - ICU Mortality | | | Study design:  RCTs, cohort studies, case control studies, case series, case reports.  Date limit: since 1980.  Limited to studies reported in English. | |
| **Summary of evidence:** | | | | | | | | | | |  |
| **Study author (ref)** | **Study type** | | **Population** | **Intervention** | | **Comparison** | | **Outcome of interest** | **Comments (GRADE level)** | |  |
| *Pepke-Zaba[212]* | Controlled trial | | Patients with severe PH; controls with normal PVR | NO 10ppm | | Intravenous prostacyclin 24mcg/h | | PVR fell after both agents, in PH and controls. SVR fell in PH patients with PGI2 but did not fall with NO. | Important early study showing NO as selective pulmonary vasodilator. MODERATE. | |  |
| *Frostell[214]* | Controlled trial | | Healthy adults n=9 | Hypoxic mixture alone (12% O2) | | Hypoxic mixture + NO 40ppm | | NO reversed hypoxic vasoconstriction without dropping SVR | LOW | |  |
| *King[213]* | Case report | | 60-yr-old woman with pulmonary fibrosis, PH, RVF, cardiac arrest | NO | | None | | NO rapidly improved oxygenation, haemodynamics, able to wean ventilatory support | VERY LOW | |  |
| *Channick[205]* | Case report | | Pulmonary fibrosis, PH, RVF | NO | | IV PGE1 | | NO led to marked reduction PVR, increased CO, Improved oxygenation. no effect on SVR. PGE1 worsened O2 desaturation. | VERY LOW | |  |
| *Schenk[215]* | Case report | | 66-yr-old woman with RVF following massive PE | NO | | None | | Dramatic improvement in haemodynamics with NO | VERY LOW | |  |
| *Fujita[218]* | Case report | | 66-yr-old man with severe RVF post-MI (post-angioplasty) with IABP in situ | NO 10ppm | | None | | NO immediately improved indices of RV failure | VERY LOW | |  |
| *Inglessis[217]* | Non-randomised controlled study | | 13 patients with cardiogenic shock post-inferior/RVMI | NO 80ppm | | Oxygen alone | | NO reduced RAP, PVR; increased CO, SVI. NO did not change SVR or PCWP. NO reduced shunt flow and improved SaO2. | MODERATE | |  |
| *Rich[219]* | Non-randomised controlled study | | 20 patients undergoing cardiac surgery or VAD insertion | NO 20ppm in 80% O2 for 6 minutes, before and after CPB | | 80% O2 alone | | NO reduced PVR and increased pO2. MAP increased in VAD patients. | NO is effective pre- and post-CPB, and in VAD-supported circs. The effect on PVR is not altered by CPB or a VAD. MODERATE. | |  |
| *Kieler-Jensen[202]* | Non-randomised controlled study | | PH after heart transplantation | NO 5, 10, 20ppm | | Intravenous SNP, PGI2, PGE1 | | Augmentation of CO highest with prostacyclin. NO was the only agent to reduce PVR while not changing SVR | Prostacyclin is the best choice of iv vasodilator following HTx, but NO should be used when systemic hypotension associated. MODERATE. | |  |
| *Macdonald[220]* | Self-controlled study | | 7 patients with PH post-LVAD insertion | NO 20ppm at time of LVAD implantation and for 24h thereafter | | No NO | | NO prevented rises in PVR at time of LVAD insertion | Rise in PVR can result in critical reduction in LVAD flow: NO should be routinely available. MODERATE. | |  |
| *Beck[221]* | Case series | | 34 high-risk cardiac surgical patients with high PVR | NO for 6-240h post op (mean 36h) | | None | | NO reduced mPAP, improved systemic MAP, enabled weaning | LOW | |  |
| *Carrier[227]* | Case reports | | 2 patients with graft failure after heart transplantation (HTx) | NO 20ppm 4-6h post-HTx | | None | | NO reduced PVR, improved CI, allowed weaning from vent support and IABP | The use of NO in comb with inotropes and IABP is suggested in primary graft failure. LOW. | |  |
| *Girardis[230]* | Case report | | 59-yr-old man with severe PH, hypotension and hypoxia after OLT (during reperfusion phase) | NO 15ppm for 16h post-operatively (with dobutamine) | | None | | PVR reduced, P/F ratio improved, extubated D4 post-op | VERY LOW | |  |
| *Molmenti[233]* | Case series | | 6 patients with severe portopulmonary hypertension (PoPH) awaiting OLT | NO 40ppm, added to by PGI2 infusion, uptitrated from 5ng/kg/h, as tolerated | | None | | PVR reduced in 5/6 patients pre-operatively with this treatment. These 5/6 had well-controlled PVR immediately post-op and survived post-op 13-24 months. Patient 6 died post-op of RVF. | If PoPH can be controlled to mPAP<35mmHg, with good RV function, OLT can be tolerated. LOW. | |  |
| *Ralley[231]* | Case report | | 75-yr-old man with protamine-induced PH with RV dilatation post-CABG | NO 40ppm (after stopping protamine, 100mg amrinone bolus, NE, dobutamine) | | None | | NO reduced PAP and increased MAP. | VERY LOW. | |  |
| *Yoshikawa[229]* | Case report | | Acute graft rejection following lung transplantation | NO | | None | | Reduces PVR, improved oxygenation, no effect on systemic haemodynamics | VERY LOW. | |  |
| *Maxey[225]* | Cohort study | | 17 cardiac surgical patients having NO for post-op PH | NO | | Pre-NO | | NO reduced mPAP and RVSWI without change in MAP | LOW | |  |
| *Capellier[216]* | Case report | | 4 patients with massive PE | NO | | None | | NO improved pulmonary haemodynamics and ga exchange | VERY LOW. | |  |
| *Trummer[228]* | Case report | | 69-yr-old woman with RVF due to massive PE | NO | | None | | PH reduced | VERY LOW | |  |
| *Takaba[224]* | Case report | | 42-yr-old man with CTEPH undergoing RV thrombectomy | NO | | None | | Perioperative NO enabled PH management and weaning | VERY LOW | |  |
| *Fattouch[223]* | Non-randomised controlled study | | 58 patients with mitral stenosis and high PVR following MVR surgery | NO 20ppm | | Inhaled PGI2 10g/min (and SNP 5-15g/min) | | Inhaled PGI2 and NO both reduced PVR and increased CO. SNP caused hypotension in 62% patients. | Inhaled agents are better than intravenous, and NO equivalent haemodynamic effects to inhaled PGI2. MODERATE. | |  |
| *Fernandez-Perez[222]* | Case report | | Acute RVF following extrapleural pneumonectomy | NO | | None | | NO improved CI by decreasing PVR without causing systemic hypotension | VERY LOW | |  |
| *Khan[226]* | Prospective randomised crossover pilot trial | | 25 HTx & LTx recipients with PH, RV dysfunction or refractory hypoxaemia | NO 20ppm for 6h then crossover to other agent | | Inhaled PGI2 20,000 ng/ml for 6h then crossover to other agent | | At 6h, both agents similarly reduced PAP, CVP, improved CI and SvO2. Neither agent affected ox index or systemic BP. | Both NO and inhaled PGI2 equivalent and effective in managing PH in thoracic transplantation. MODERATE. | |  |
| *Bender[235]* | Self-controlled study | | 13 cardiac surgical patients with persistent PH hypoxemia post-CPB | Pre-NO | | Post-NO 20ppm | | PVR/SVR reduced and increased oxygenation index | LOW | |  |
| *Snow[234]* | Non-randomised controlled study | | 2 groups of patients: 1. following MVR surgery with pre-existing PH and 2. CABG surgery (without PH) | NO 40ppm in group with PH | | NO 40ppm in group without PH | | NO reduced PVR in patients with pre-existing PH; NO did not change haemodynamics in patients with normal PAP | LOW. | |  |
| *Solina[236]* | Prospective randomised controlled study | | 45 cardiac surgical patients with PH on separation from CPB | NO 20ppm, 40ppm | | Milrinone (intravenous) | | 40ppm NO increased RVEF compared to the lower dose and to MIL. Milrinone group needed more vasopressors and were more tachycardic. | MODERATE. | |  |
| *Solina[238]* | Prospective, randomised, non-blinded study | | 62 consecutive cardiac surgery patients with PH evident before anaesthesia | 10ppm, 20ppm, 30ppm,or 40ppm NO at end of CPB | | Milrinone alone control | | Percentage drop in PVR not sig different among the NO groups. | Doses >10ppm not justified in this population. MODERATE. | |  |
| *Solina[237]* | Comparative study of 2 subpopulations of PH (severe vs less severe) | | 30 consecutive cardiac surgical patients with PH prior to anaesthesia. | PVR 125-300 dynes/s/cm5, given NO 30ppm on separation from CPB | | PVR >300 dynes/s/cm5, given NO 30ppm | | Patients with higher baseline PVR had greater reduction with NO | NO affects cardiac surgical patients with high PVR more than low PVR. LOW. | |  |
| *Fattouch[239]* | RCT | | 58 cardiac surgical patients with mitral stenosis and PH (PVR>250 dynes/s/cm5). PAC; TOE. | NO or Inhaled PGI2 (5 minutes before weaning from CPB) | | Intravenous vasodilators | | Both agents reduced PVR and increased CI and RVEF compared to controls. Effects were sustained. Inhaled drug groups had shorter weaning from CPB, shorter intubation time, and shorter ICU stay. | HIGH | |  |
| *Healy[240]* | Case report | | 56-yr-old man with acute MR undergoing emergency surgery. post MVR hypotension due to RVF and severe PH. | NO | | None | | NO enabled rapid improvement and weaning from CPB | VERY LOW | |  |
| *Gerlach[248]* | RCT | | 40 patients with ARDS | Dose-response of long-term NO: continuous dose (10ppm), then challenged with 10-100ppm NO | | Conventional therapy (no continuous NO); then challenged with 10-100ppm NO | | Shift in dose-response to NO from Day 0 to day 4. Oxygenation worsened at high doses after 4 days NO | HIGH | |  |
| *Rossaint[241]* | Non-randomised controlled study | | 9 consecutive ARDS patients | NO 5-20ppm for 3-53 days | | Intravenous PGI2 | | NO 18ppm reduced mPAP and improved oxygenation index. CO and MAP unchanged. IV PGI2 also reduced mPAP but increased shunting, worsened oxygenation and MAP. | NO reduces PAP and increases arterial oxygenation without dropping MAP. MODERATE. | |  |
| *Bigatello[246]* | Non-randomised controlled study | | 13 patients with severe ARDS | NO 5-40ppm | | 7 of the 13 continued to breathe 2-20ppm NO for 2-27 days. | | NO reduced mPAP and PVR in a dose-related fashion, without changing MAP. Oxygenation improved without clear dose-response effect. Also effective without evidence of tachyphylaxis in long-term group. | LOW. | |  |
| *Chiche[244]* | Case report | | Post-pneumonectomy ARDS and PH with RVF | NO | | None | | NO improved haemodynamics and oxygenation | VERY LOW | |  |
| *Fierobe[242]* | Self-controlled study | | 13 patients with severe ARDS and PH (mPAP>30mmHg). PAC. | Pre-NO 5ppm | | Post-NO | | NO dropped PAP and PVR; increased RVEF, improved oxygenation. No relationship between improved oxygenation and PVR. | LOW. | |  |
| *Benzing[245]* | Case report | | Patient with ARDS and acute RVF (echo) | NO (3 days) | | None | | NO treatment increased CO by 32% in dose-dependent manner, until RV function had improved. | VERY LOW | |  |
| *Bhorade[79]* | Self-controlled study | | 26 ARDS patients with acute RV dysfunction on echo | Pre-NO | | Post-NO (10ppm increments) until max effect. mean 35ppm needed | | NO increased CO, SV, SvO2, with reduced PVR, and on discontinuation all returned to baseline. | Most patients had a response at 40ppm or less. no mortality endpoints. MODERATE. | |  |
| *Romberg-Camps[243]* | Case report | | ARDS with RVF in eclampsia and staph sepsis | NO | | None | | NO stabilised haemodynamics | VERY LOW | |  |
| *Hsu[247]* | Self-controlled study | | 32 ARDS patients | NO | | Increasing concentrations (1, 5, 10, 20, 40ppm) | | Oxygenation improved in lower ranges (1-20ppm); PVR reduced in higher ranges (1-40ppm) | Optimal doses for improving oxygenation and reducing PAP differ. LOW. | |  |
| *Manktelow[252]* | Retrospective self-controlled study. Who gets a ‘useful response’ to NO? (‘useful response’ defined as a 20% improvement in P/F ratio, or 20% fall in PVR) | | 88 ARDS patients | Pre-NO | | Post-NO | | 58% had a clinically sig response to NO. Those with initial favorable response maintained it at 48h. Patients with additional sepsis less likely to respond favourably. | LOW. | |  |
| *Taylor[250]* | Multicentre RCT | | 385 patients with moderately severe ARDS without sepsis | NO 5ppm for 28 days | | Placebo (nitrogen gas) | | Significant increase in pO2 at 48h. No difference in ventilation requirement or mortality. | HIGH. | |  |
| *Sokol[254]* | Meta-analysis of 5 RCTs | | 535 patients with ARDS | NO | | No NO | | No difference in vent-free days or mortality. Oxygenation improved in first 4 days. |  | |  |
| *Troncy[255]* | RCT | | ARDS due to sepsis (25 of 30) | NO 0.5-40ppm daily | | No NO | | NO improved oxygenation but made no impact on mortality | HIGH. | |  |
| *Michael[256]* | RCT | | ARDS patients | NO for 72 | | Conventional treatment without NO | | NO improved oxygenation very early, but only for up to 24h. | MODERATE. | |  |
| *Dellinger[249]* | Multicentre RCT | | 177 ARDS patients within 72h of diagnosis, excluding sepsis | NO varying doses up to 80ppm | | Nitrogen | | NO well tolerated. Small reduction PAP. | HIGH. | |  |
| *Lundin[257]* | Multicentre RCT | | 268 patients with EARLY ALI | NO (incrementing to 40ppm) to increase PaO2 by 20% within 96h | | Conventional therapy without NO | | No difference in reversal of ALI between NO-responders and non-responders | Oxygenation improved but no impact on outcomes. HIGH. | |  |
| *Taylor[250]* | Multicentre RCT | | ARDS, excluding sepsis | NO 5ppm | | Nitrogen | | Short-term improved oxygenation; no other outcomes improved. Small reduction PAP. | HIGH. | |  |
| *Adhikari[253]* | Meta-analysis of 12 trials | | 12 trials assigning 1237 patients with ALI/ARDS | NO | | No NO | | NO improved oxygenation on day 1, lasting until day 4. No effect on mPAP. No effect on mortality or ventilator-free days. | HIGH | |  |
| *Brett[367]* | Controlled non-randomised study | | 26 patients with established ARDS | NO responders | | NO non-responders | | No difference in 2 groups in terms of HRCT, BAL inflammation, oxygenation, PVR OR survival. | MODERATE. | |  |
| *Vater[258]* | Case report | | 49-yr-old man with end-stage cirrhosis and PoPH. PAC.TOE. | NO (10-40ppm) in addition to iv PGI2 prior to anaesthesia | | None | | PAP dropped as NO concentration increased. | VERY LOW | |  |
| *Flondor[259]* | Case report | | Acute PH following PEA surgery | NO added to inhaled iloprost | | Inhaled iloprost alone | | Combination of inhaled agents were effective at managing post-op PH crisis with additive effects compared to iloprost alone | VERY LOW | |  |
| *Lepore[260]* | Case series | | 11 patients with PH due to left-sided heart disease | NO (80ppm), sildenafil (50mg), combination | | Baseline | | Sildenafil alone decreased PVR (12%), SVR (13%) (but did not change MAP), PCWP; increased CI (14%). Combined NO + sildenafil decreased PVR by 50%, SVR by 24%, CI increased by30%. Sildenafil prolonged effect of NO. | Sildenafil improves CO by balanced pulmonary and systemic vasodilatation, and augments and prolongs haemodynamic effects of NO. LOW. | |  |
| *Atz[263]* | Case series | | 9 patients with post-op PH following repair of total anomalous pulmonary venous connection (TAPVC) | NO 80ppm for 15 minutes; 5 patients had prolonged treatment with 20ppm. | | Baseline | | PVR fell with NO. After prolonged treatment, PAP transiently increased in all patients when NO stopped. | Important to appreciate this potential rebound PH. LOW. | |  |
| *Lavoie[262]* | Case report | | 4 patients with hypoxic respiratory failure. | NO | | None | | When NO stopped, PH and oxygenation worsened. | VERY LOW | |  |
| *Atz[267]* | Case report | | 3 cases of PH following TAPVC surgery | Sildenafil | | None | | Sildenafil attenuated rebound PH in this setting | VERY LOW | |  |
| *Mychaskiw[270]* | Case report | | Cardiac surgery | Sildenafil | | None | | Sildenafil blunted rebound PH following withdrawal of NO and milrinone. no adverse effects on SVR. | VERY LOW | |  |
| *Trachte[266]* | Retrospective self-controlled study | | 8 patients with post-op PH weaning from NO and intravenous vasodilators | Pre-oral sildenafil | | Post–oral sildenafil | | 1 oral dose sildenafil reduce PVR (49%) and had no effect on CI, MAP or SVR. | Sildenafil effectively treated post-op PH and allowed weaning of inhaled and iv pulmonary vasodilators. LOW. | |  |
| *Namachivayam[268]* | RCT | | 30 ventilated children receiving at least NO 10ppm | Oral sildenafil (0.4mg/kg) | | Placebo | | Rebound PH (>20% inc in PAP) occurred in 70% placebo but in none of the sildenafil patients | Sildenafil prophylaxis prevented rebound PH after NO withdrawal, and reduced duration of mechanical ventilation.  MODERATE. | |  |
| *Giacomini[265]* | Case report | | 66-yr-old ARDS and refractory PH on NO | Oral vardenafil (10mg and 5mg) to aide weaning from NO | | None | | 10mg caused hypotension. 5mg decreased PVR without systemic hypotension; and allowed NO withdrawal & weaning from mechanical ventilation | VERY LOW | |  |
| *Klodell[269]* | Case series | | 10 patients with LVADs using NO | Oral sildenafil (and dobutamine and milrinone) | | Baseline | | Sildenafil dropped PAP within 90 minutes. No change in MAP or SVR or HR. All patients weaned from NO (and from mechanical vent) without recurrent PH. | VERY LOW | |  |
| *PROSTANOIDS* |  | |  |  | |  | |  |  | |  |
| *Rubin[368]* | Self-controlled study | | 7 patients with PAH. RHC. | Incremental PGI2 iv (2-12ng/kg/min) | | Baseline | | PGI2 reduced PAP, increased CO, reduced MAP | Important early study showing acute reduction in PVR in PAH and improved CO. MODERATE. | |  |
| *Barst[274]* | Multicentre RCT | | 81 patients with severe PAH (NYHA III-IV) | PGI2 infusion over 12 weeks | | Conventional therapy for PAH | | PGI2 improved exercise capacity and haemodynamics | HIGH | |  |
| *Olschewski[278]* | Case report | | 45-yr-old woman with decompensated PAH | Iloprost inhaled; NO | | intravenous prostacyclin | | Both inhaled iloprost and NO decreased PVR, but only iloprost dropped mPAP. Both improved CO and oxygenation. IV PGI2 not tolerated. | VERY LOW. | |  |
| *Hoeper[277]* | Self-controlled study | | 35 patients with PAH | Inhaled iloprost and NO | | Baseline | | Both increased CO, SvO2, SV; both reduced PAP and PVR. Iloprost more effective than NO. | Inhaled iloprost more potent pulmonary vasodilator than NO in PAH. MODERATE. | |  |
| *Hoeper[276]* | Self-controlled study | | 24 patients with PAH | Inhaled iloprost 100-150mcg/d for 1y | | Baseline | | Iloprost had sustained effects on exercise capacity and haemodynamics | MODERATE | |  |
| *Olschewski[275]* | Multicentre RCT | | 203 patients with PAH | Inhaled iloprost (2.5-5mcg 6-9 times/day, i.e. 30mcg/day) | | Inhaled placebo | | Iloprost improved haemodynamics, improved NYHA class, dyspnoea and quality of life. | HIGH. | |  |
| *Kieler-Jensen[279]* | Comparative study | | Patients post-HTx | IV prostacyclin | | IV GTN, SNP | | PGI2 improved CO and SV the best. PVR/SVR no different between agents | LOW | |  |
| *Ocal[18]* | RCT | | 68 CABG patients with acute PH due to protamine | IV PGI2, NE and dopamine | | IV GTN, NE and dopamine | | PVR decreased with PGI2 compared to controls | IV PGI2 effective at treating protamine-induced PH. MODERATE. | |  |
| *D'Ambra[280]* | Case series | | 5 patients with post-CPB PH | High dose PGE1 (30-150ng/kg/min) with NE | | None | | All patients had rapid pulmonary vasodilatation with improved RV function | NE important to prevent drop in MAP. VERY LOW. | |  |
| *Vincent[281]* | Case series | | 18 patients with RVF post-HTx | PGE1 infusion for up to 7 days, with NE into LA catheter | | Baseline | | PGE1 reduced PVR and increased CI. MAP was stable. | NE important to prevent drop in MAP. LOW. | |  |
| *Schmid[283]* | Randomised crossover study | | 14 patients post cardiac surgery with severe PH but preserved RV function | NO 40ppm | | PGE1; GTN IV | | All agents reduced PVR. NO caused selective pulmonary vasodilatation; while the systemic agents caused systemic hypotension. PGE1 and NO increased CI compared to GTN. | Severe PH does not always imply RV dysfunction. In these patients with preserved RV function, NO was no better than IV PGE1 in terms of CI and RV performance.  MODERATE. | |  |
| *Elliott[284]* | Case report | | Persistent PH following MVR for mitral stenosis | IV PGI2 | | None | | Successful management of post-op PH | VERY LOW | |  |
| *Radovancevic[282]* | Randomised crossover trial | | 19 HTx candidates with PH | IV PGE1 | | NO | | Comparable effect s on PVR reduction. More systemic hypotension with PGE1. | MODERATE. | |  |
| *Haraldsson[285]* | Case series | | 9 patients with post-op PH and RVF after cardiac surgery or HTx | Inhaled PGI2 at 2.5, 5 and 10mcg/ml | | Baseline | | Inhaled PGI2 reduced PVR, without changing SVR. RV performance improved. | LOW | |  |
| *Schroeder[288]* | Case report | | 4 patients with post-CABG PH and RVF | Inhaled PGI2 | | None | | Inhaled GI2 effectively reduced PVR, inc’d CI, allowed successful weaning and extubation post-op | VERY LOW | |  |
| *Hache[286]* | Large case series | | 35 patients with PH in ICU (mixed group) | Inhaled PGI2 | | None | | Achieved selective pulmonary vasodilatation and improved oxygenation in most. Small number got systemic hypotension. | LOW. | |  |
| *Lowson[289]* | Case report | | 63-yr-old woman post aortic and mitral valve surgery with PH and RVF post-CPB | Inhaled PGI2 50ng/kg/min (added to milrinone, NE, GTN which were preventing her weaning from CPB due to RVF) | | None | | Allowed successful weaning from CPB. Continued for 4/7 post op; no tolerance or systemic effects | VERY LOW | |  |
| *De Wet[287]* | Self-controlled study | | 126 patients with PH, refractory hypoxia and RV dysfunction post-cardiothoracic surgery | Inhaled PGI2 for an average of 45.6h | | Baseline | | Reduced PAP without reducing MAP | LOW | |  |
| *Fattouch[223]* | Prospective DB randomised study | | 58 patients with mitral stenosis and high PVR following MVR surgery | NO 20ppm | | Inhaled PGI2 10g/min (and SNP 5-15g/min) | | Inhaled PGI2 and NO both reduced PVR and increased CO. SNP caused hypotension in 62% patients. | Inhaled agents are better than intravenous, and NO equivalent haemodynamic effects to inhaled PGI2. MODERATE. | |  |
| *Sablotzki[295]* | Non-randomised study | | 14 HTx candidates with elevated PVR (>180dynes/sec/cm5) | Inhaled iloprost (50mcg) | | NO (5, 10, 30ppm) | | Both inhaled agents reduce PVR. Iloprost was more effective at reducing mPAP than 10 and 30ppm NO. | LOW. | |  |
| *Theodoraki[296]* | Self-controlled study | | 12 patients with persistent PH post CPB | Inhaled iloprost for 20min (0.2mcg/kg total) | | Baseline | | Reduced PVR/SVR. Echo indices RV function improved. no adverse MAP effects | Suitable for initial management of perioperative PH to improve RV performance. LOW. | |  |
| *Tissieres[294]* | Case report | | Patient with end-stage CF and PH | Inhaled iloprost | | None | | Successful use and bridging to LTX | VERY LOW | |  |
| *Kramm[290]* | RCT | | 22 patients with persistent PH post-PEA | Single dose 25mcg nebulized iloprost | | Saline control | | Iloprost reduced PVR, increased CI. Oxygenation unchanged. | MODERATE. | |  |
| *Baysal[293]* | Non-randomised study | | 18 patients with PH undergoing valve surgery, pre and post CPB | Intravenous iloprost | | Intravenous GTN | | Iloprost 1.25-2.5ng/kg/min reduced PVR better than GTN 0.5-1 mcg/kg/min. iloprost increased CO more than GTN. | LOW. | |  |
| *Yurtseven[292]* | RCT | | 100 patients with PH undergoing MVR | Inhaled iloprost | | Inhaled GTN | | Both reduced PVR. PVR even lower with iloprost. MAP higher with iloprost. Iloprost increased CO and SV. | Iloprost more effective than inhaled GTN in MVR-related post-op PH. MODERATE | |  |
| *Rex[291]* | RCT | | 20 patients with chronic PH undergoing MVR. PAC, TOE. | Inhaled iloprost, given during weaning from CPB | | Intravenous GTN | | Iloprost decreased PVR and increased RVEF, SVI, CPB weaning successful on first attempt (c.f. GTN where 3 failed wean). | Inhaled iloprost superior to iv GTN reduced RV afterload and moderately improving RV-pump performance. MODERATE. | |  |
| *Khan[226]* | Prospective randomised crossover pilot trial | | 25 HTx & LTx recipients with PH, RV dysfunction or refractory hypoxaemia | NO 20ppm for 6h then crossover to other agent | | Inhaled PGI2 20,000 ng/ml for 6h then crossover to other agent | | At 6h, both agents similarly reduced PAP, CVP, improved CI and SvO2. Neither agent affected ox index or systemic BP. | Both NO and inhaled PGI2 equivalent and effective in managing PH in thoracic transplantation. MODERATE. | |  |
| *Winderhalter[297]* | RCT | | 46 patients with pre-existing PH undergoing cardiac surgery | Inhaled iloprost | | NO (at end of CPB) | | Both reduced PVR with sig rise in CO, with iloprost sig more effective. | MODERATE | |  |
| *Webb[298]* | Case report | | Severe PH due to acute-on-chronic massive PE | Inhaled PGI2 | | Conventional medical therapy | | Transient improvement in pulmonary haemodynamics and gas exchange | VERY LOW | |  |
| *Radermacher[300]* | Self-controlled study | | 8 patients with ARDS. PAC. | PGI2 infusion | | Baseline | | PAP fell; CI increased with inc SV, reduced PVR. In patients with subnormal RVEF, PGI2 improved RVEF. Increased venous admixture but PaO2 was unchanged as DO2 increased. | IV PGI2 improves RV parameters when baseline RV function is depressed. LOW. | |  |
| *Walmrath[303]* | Self-controlled study | | 3 patients with severe ARDS | Aerosolized PGI2 (17-50ng/kg/min) | | Baseline | | 30% reversible drop in PVR; Systemic pressure slight drop; P/F ratio inc’d mainly due to reduced VQMM | LOW. | |  |
| *Van Heerden[302]* | Self-controlled study | | 9 patients with severe ARDS | Aerosolized PGI2 (0-50ng/kg/min) | | Baseline | | Improved P/F ratio; no effect on systemic or pulmonary artery pressures, nor on platelet aggregation. | LOW. | |  |
| *Zwissler[301]* | Non-randomised study | | Severe ARDS patients | Inhaled PGI2 (1, 10 and 25ng/kg/min) | | NO (1, 4, 8ppm) | | Both induce selective pulmonary vasodilatation and increase PaO2. | LOW. | |  |
| *Van Heerden[304]* | Case report | | 2 Severe ARDS patients | Aerosolized PGI2 (30-40ng/kg/min) | | Baseline | | Dose >30ng/kg/min improved severe hypoxaemia without systemic hypotension. | VERY LOW | |  |
| *Meyer[305]* | Self-controlled study | | 15 patients with ARDS | IV PGE1 (41mcg/h) | | Baseline | | P/F ratio improved. Reduction in mPAP, CI, HR NS. No change in systemic BP. | LOW | |  |
| *Kuhlen[306]* | Case report | | 1 patient with severe ARDS | Combined iv PGI2 (4ng/kg/min) and NO (10ppm) | | Baseline | | Marked drop in PAP and inc in CO compared o drugs on own. Oxygenation worse with IV PGI2 alone; improved with the combination. | VERY LOW | |  |
| **PDE5 inhibitors** |  | |  |  | |  | |  |  | |  |
| *Preston[308]* | Non-randomised controlled study | | 20 patients with PAH | Oral sildenafil | | Compared to NO, both, and IV PGI2 | | Potent acute fall in PVR, increase in CO. Effects potentiated with NO. | LOW. | |  |
| *Michelakis[309]* | Non-randomised controlled study | | 13 consecutive patients with PH. | Sildenafil | | NO 80ppm | | Similar drop in PVR with both agents; Combination potentiated effect. sildenafil + NO increased CI; NO did not.MAP similar between groups. | A single oral dose of sildenafil is as effective a pulmonary vasodilator as NO. Sildenafil may be superior as increases CO and does not increase PAWP. MODERATE. | |  |
| *Madden[318]* | Case report | | 2 patients with post-cardiac surgery PH (both patients had pre-existing PH) | Oral sildenafil: case 1 post-induction of anaesthesia; case 2 after coming off CPB | | None | | Significantly reduced PVR with a rise in systemic BP | VERY LOW | |  |
| *Shim[317]* | RCT | | 53 patients with PH undergoing heart valve surgery | Oral sildenafil 50mg prior to induction of anaesthesia | | Placebo | | PVR lower in sildenafil group at 30mins; without any change in SVR or MAP | MODERATE | |  |
| *Klodell[269]* | Case series | | 10 patients with LVADs using NO | Oral sildenafil (and dobutamine and milrinone) | | Baseline | | Sildenafil dropped PAP within 90 minutes. No change in MAP or SVR or HR. All patients weaned from NO (and from mechanical vent) without recurrent PH. | LOW | |  |
| *De Santo[316]* | Case series | | 13 patients with RV dysfunction following HTx (with pre-existing PH). PAC. | Sildenafil | | None | | Within 72h, acute RV dysfunction resolved; PVR fell; no sig systemic side effects. | LOW. | |  |
| *Trachte[266]* | Retrospective self-controlled study | | 8 patients with post-op PH weaning from NO and intravenous vasodilators | Pre-oral sildenafil | | Post–oral sildenafil | | 1 oral dose sildenafil reduce PVR (49%) and had no effect on CI, MAP or SVR. | Sildenafil effectively treated post-op PH and allowed weaning of inhaled and iv pulmonary vasodilators. LOW. | |  |
| *Atz[267]* | Case report | | 3 cases of PH following TAPVC surgery | Sildenafil | | None | | Sildenafil attenuated rebound PH in this setting | VERY LOW | |  |
| *Namachivayam[268]* | RCT | | 30 ventilated children receiving at least NO 10ppm | Oral sildenafil (0.4mg/kg) | | Placebo | | Rebound PH (>20% inc in PAP) occurred in 70% placebo but in none of the sildenafil patients | Sildenafil prophylaxis prevented rebound PH after NO withdrawal, and reduced duration of mechanical ventilation.  MODERATE. | |  |
| *Lepore[260]* | Case series | | 11 patients with PH due to left heart disease | Oral sildenafil 50mg | | NO 80ppm, or both | | Sildenafil alone red’d PVR; SVR; PAOP; incr’d CI. NO-Sildenafil combination all similar greater magnitude effects. | Neither sildenafil alone or with NO reduced systemic BP. VERY LOW. | |  |
| *Ghofrani[321]* | RCT | | 16 patients with PH due to lung fibrosis | Oral sildenafil 50mg | | NO 10-20ppm;  IV PGI2 max tolerable dose (mean 8ng/kg/min) | | PVR fell with all 3 agents. PVR/SVR only fell with NO and sildenafil. PGI2 increased VQMM and decreased PaO2; NO and sildenafil maintained VQ matching with raised PaO2. | Sildenafil caused preferential pulmonary vasodilatation and improved gas exchange in severe lung fibrosis and secondary PH. HIGH | |  |
| *Ng[322]* | Case report | | Patient with severe secondary PH + RV dysfunction | Oral sildenafil added to NO | | None | | Reduced mPAP, PVR maintained; allowed weaning of CVS support | VERY LOW | |  |
| *Botha[324]* | RCT | | 30 patients undergoing HTx assessment by RHC | Milrinone 0.05mg/kg bolus | | Sildenafil high (0.43mg/kg) or low (0.05mg/kg) dose | | Doses of sildenafil no different. Both agents reduced PVR and SVR similarly. Milrinone reduced PAOP and mPAP more. Greater increases in CI seen in those with higher baseline PAWP. | MODERATE | |  |

**Abbreviations**: NO nitric oxide, HTx heart transplantation, LTx lung transplantation, CPB cardiopulmonary bypass, OLT orthotopic liver transplantation, VAD ventricular assist device, SNP sodium nitroprusside, MVR mitral valve replacement, MS mitral stenosis, VQ ventilation-perfusion, BAL bronchoalveolar lavage,, RHC right-heart catheterisation

**Structured clinical question 8:** Which mechanical therapies are effective in patients with pulmonary vascular dysfunction?

**PICO 8: Mechanical support in pulmonary vascular dysfunction**

| **Patient Population** | | **Intervention** | | | **Comparison** | | **Outcomes** | | | **Comments** | |
| --- | --- | --- | --- | --- | --- | --- | --- | --- | --- | --- | --- |
| Adult ICU patients at risk of developing RV dysfunction / failure as a result of a period of elevated PVR.  Including:   - Adults receiving post-operative care for cardiothoracic and transplantation surgery - Patients with known pre-existing PH undergoing surgery (including obstetric procedures) - Other clinical subgroups at risk of PVD including acute PE, ARDS/ALI, sepsis. | | The clinical effectiveness of mechanical support in PVD  Search terms:*:*   - RVADS - ECMO - IABP - Atrial septostomy | | | ‘Standard usual care’ in cardiothoracic ICU | | - Effects on RV function (measures including SV, RVEF,CO, MAP) - Effects on PVR - Morbidity: any adverse events - Effects on CPB weaning - ICU length of stay - ICU Mortality | | | Study design:  RCTs, cohort studies, case control studies, case series, case reports.  Date limit:  since 1980.  Limited to studies reported in English. | |
| **Summary of evidence: Mechanical devices** | | | | | | | | | | |  |
| **Study author (ref)** | **Study type** | | **Population** | **Intervention** | | **Comparison** | | **Outcome of interest** | **Comments (GRADE level)** | |  |
| *Giesler[338]* | Case report | | 57-yr-old woman post-inferior MI with cardiogenic shock due to severe RVF despite reperfusion and IABP | RVAD insertion | | None | | Improved MAP to allow withdrawal of inotropes | VERY LOW | |  |
| *Fonger[339]* | Case report | | Severe PH following LTx. | RVAD and intravenous PGE1 | | None | | Managed RVF | VERY LOW | |  |
| *Nagarsheth[340]* | Case report | | Severe PH and RV failure due to presumed amniotic fluid embolism | RVAD | | None | | Successful management | VERY LOW | |  |
| *Strueber[343]* | Case report | | 4 patients with cardiogenic shock due to end-stage PH (including PVOD). | Pumpless lung-assist device to unload the RV (from PA trunk to LA) | | None | | All patients successfully bridged to transplantation | VERY LOW | |  |
| *Jones[347]* | Case report | | 41-yr-old man with critical MS for emergency MVR. difficult to wean from CPB due to severe PH (61/45mmHg), poor pulmonary blood flow (CO2 hard to eliminate) | ECMO (all cases veno-arterial); 2l/min | | None | | PAPs decreased from 61 to 40mmHg, PVR reduced, weaned CPB and from IPPV. | VERY LOW. | |  |
| *Chan[346]* | Case report | | Patient with PAH at transplantation. | ECMO for 24h after transplantation. | | None | | Reduction in pulmonary blood flow minimised reperfusion oedema of graft. Smooth recovery. | VERY LOW | |  |
| *Satoh[345]* | Case report | | 33-yr-old with PAH having termination of pregnancy (CS under GA) at 18 weeks | ECMO 1.5l/min set up preoperatively | | None | | PAP gradually decreased while MAP unchanged throughout GA. Despite NO & PGI2, ECMO unweanable. Patient died on D19 of RVF. | VERY LOW | |  |
| *Felton[344]* | Case report | | 43-yr-old man with acute PH and RVF following aggregate anaphylaxis | ECMO | | None | | ECMO effective | VERY LOW | |  |
| *Gregoric[348]* | Case report | | Patient with PH at embolectomy and post-operative RVF, RVAD inserted. | ECMO also used as bridge to transplant as pulmonary haemorrhage++ | | None | | ECMO for 10 days was effective bridge to transplant | VERY LOW | |  |
| *Hsu[349]* | Case report | | 23-yr-old woman with severe PAH and SLE | ECMO | | None | | ECMO successful bridge to transplant | VERY LOW | |  |
| *Berman[350]* | Comparative study | | 127 PTE surgical patients, 5.5% requiring post-operative VA-ECMO | ECMO | | Patients not requiring ECMO | | 73% successfully weaned from ECMO. Worse pre-op haemodynamics (higher mPAP; PVR) in group needing ECMO | LOW | |  |
| *Szocik[353]* | Case report | | 54-yr-old woman with acute PH and RVF due to massive PE during OLT for PBC | ECMO (set up within 45 minutes) | | None | | Immediate improvement in haemodynamics | VERY LOW | |  |
| *Deehring[351]* | Case report | | 17-yr-old female with massive PE and RA thrombus. CVS collapse: for embolectomy | ECMO | | None | | ECMO enabled stabilisation for transfer for embolectomy surgery | VERY LOW | |  |
| *Haller[352]* | Case report | | 36-yr-old with acute particle embolus (causing PH and RVF) during AVM coil embolization | ECMO | | None | | Stabilised haemodynamics | VERY LOW | |  |
| *Arlt[354]* | Case report | | 27-yr-old with postpartum PE and cardiac arrest | ECMO (handheld) | | None | | ECMO allowed stabilisation prior to surgical embolectomy | VERY LOW | |  |
| *Gold[355]* | Case report | | Patient with PE and severe rheumatic valvular heart disease at embolectomy | Pulmonary artery balloon pump (PABP) | | None | | PABP allowed CPB discontinuation and post-CPB haemodynamic support | VERY LOW | |  |
| *Arafa[356]* | Self-controlled clinical trial | | 12 patients with high PVR & RVF after HTx. Does IABP alleviate RV dysfunction after HTx? | IABP | | Baseline values | | IABP improved CI, SvO2; reduced PCWP, CVP, PAP. All weaned. | IABP was effective in low CO with RV allograft failure. LOW. | |  |
| *Rothman[358]* | Case series | | 12 patients with PH (75% PAH) | Atrial septostomy | | Baseline | | RAP decreased; O2 saturations decreased; CI increased.50% patients had clinical improvement. | Maybe a useful bridge to transplantation. LOW | |  |
| *Rich[359]* | Case series | | 6 patients with severe end-stage PAH (1983-95). Compared to 2 previous major series | Atrial septostomy | | Baseline | | Reduced RAP, improved tissue oxygenation at expense of SaO2. 3 procedure-related deaths in most severe patients: hypotension, desaturation and cardiac arrest. | Suggest do not attempt in setting of severe RVF with high RAP and low CI.  VERY LOW | |  |
